# Enantiomeric Excess Bupivacaine in a Lavender Oil NLC Tested in a Melanoma Model: Prolonged Release and Anticancer Effect

**DOI:** 10.1021/acs.molpharmaceut.5c00254

**Published:** 2025-05-01

**Authors:** Gabriela Geronimo, Gustavo H. Rodrigues da Silva, Ludmilla D. de Moura, Fabíola V. de Carvalho, Talita C. Mendonça, Laura B. Olivo, Bibiana Verlindo de Araújo, Teresa C. Dalla Costa, Luccas Lavareze, Fernanda V. Mariano, Eneida de Paula

**Affiliations:** † Department of Biochemistry and Tissue Biology, 28132 Institute of Biology, University of Campinas—UNICAMP, Campinas, SP 13083-862, Brazil; ‡ Brazilian Biosciences National Laboratory, Brazilian Center for Research in Energy and Materials, Campinas, SP 13083-970, Brazil; § Pharmaceutical Sciences Graduate Program, Faculty of Pharmacy, 28124 Federal University of Rio Grande do Sul—UFRGS, Porto Alegre, RS 90610-000, Brazil; ∥ Department of Pathology, Faculty of Medical Sciences, 67791 UNICAMP, Campinas, SP 13083-888, Brazil

**Keywords:** nanostructured lipid carriers, drug delivery, bupivacaine, melanoma, lavender oil

## Abstract

Recent studies have highlighted the potential of local anesthetics (LA) as adjuvants in cancer treatment, specifically by increasing survival rates when used in surgical excisions. However, the clinical use of LA is restricted due to their systemic toxicity. The development of drug delivery systems could address this issue and advance the utilization of these molecules. In this research, we explored the pharmacokinetics (using microdialysis probes) and antitumor properties of a nanostructured lipid carrier (NLC) formulation containing the commercially available enantiomeric excess form of bupivacaine (BVC_S75_). This NLC was prepared with lavender oil (NLC-L-BVC), an excipient with inherent antitumor properties. We compared this formulation to a control (NLC-BVC) using synthetic lipids. Pharmacokinetic assessments of the NLCs confirmed the sustained release of BVC_S75_ within the tumor, characterized by a reduced elimination rate constant and longer half-life (∼6×). The encapsulation of BVC_S75_ within nanoparticles (whether natural or synthetic) enhanced its effectiveness in treating the primary tumor, resulting in the inhibition of tumor growth (70% with NLC-L-BVC and 72% with NLC-BVC), outperforming free BVC (17% inhibition). However, the association of lavender oil with BVC_S75_ in an NLC did not yield synergistic properties. Furthermore, all BVC_S75_ treatments (whether free or encapsulated) improved animal survival rates. These findings confirm that encapsulation of bupivacaine in NLC can prolong drug action at the local site, contributing to improved local antitumor therapy while mitigating systemic effects.

## Graphical abstract

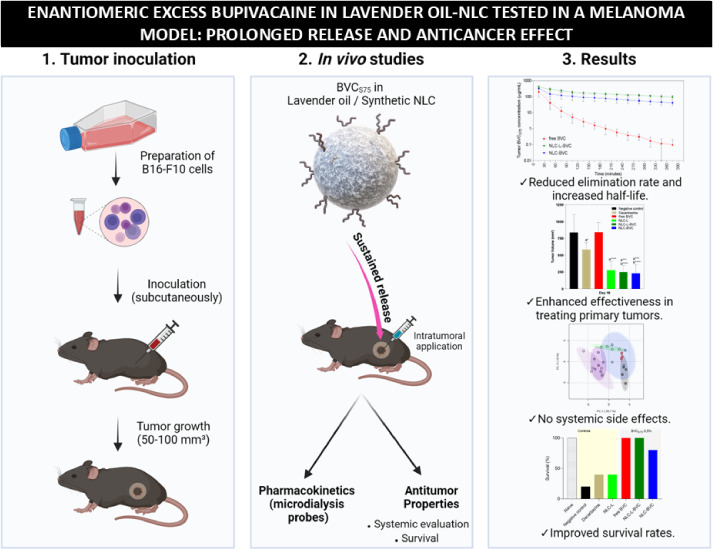

## 1 Introduction

Local anesthetics (LA) are drugs that reversibly inhibit the conduction of nerve impulses, resulting in complete analgesia and motor blockade.^1,2^ These molecules have an established use in clinical and surgical procedures, aiding in the management of acute pain and inflammation.^3^ However, an accumulating body of evidence suggests that different classes of anesthetics can elicit either pro- or antimetastatic effects, depending on the type of cancer cell, dosage, and administration protocol.^3–8^ By evaluating LA mechanisms of action in tumor cells, therapists can establish methodologies for selecting an anesthetic strategy and clinical regimen with the best antitumor properties and increased patient survival.^7–10^ In clinical cancer treatment, the adjuvant effect of LA is linked to their immunomodulatory action, which enhances antitumor immunity by reducing pro-inflammatory cytokines and stress hormones.^3,11^ Furthermore, LA help minimize surgical inflammation and establish an unfavorable environment for tumor proliferation.^12^

Recent reports have unveiled that lidocaine, a local anesthetic (LA) from the aminoamide family, has antineoplastic properties akin to oncology treatments when directly applied to malignant lesions,^3,5^ affecting the viability, migration, and invasiveness of cancer cells.,^3,5,13^ Indeed, preclinical studies have demonstrated that aminoamide LA exhibit antiproliferative, antimetastatic, and pro-apoptotic activities in various types of tumor cells^6,13,14^ through mechanisms such as the suppression of protein translation, cytoskeletal remodeling, autophagy induction, cell cycle arrest, aerobic glycolysis rate reduction, and increased expression of caspase-3.^14–17^ These interconnected mechanisms through different pathways collectively contribute to the induction of cancer cell apoptosis.^3,6,10,11^

Bupivacaine (BVC) is a long-acting aminoamide LA widely used in prolonged surgical procedures,^18,19^ although its cardiotoxicity is also well-documented in clinical practice.^20–22^ Due to the chiral carbon present in its piperidine ring, BVC is a racemic mixture, of which the levorotatory form, S(−), has a lower degree of toxicity to the cardiovascular and central nervous systems, while the dextrorotatory, R­(+), form is more potent.^23,24^ This is due to the stereoselectivity of its enantiomers, where the R­(+) isomer exhibits greater binding to the cardiac Na channel than the S(−) isomer, increasing the propensity for cardiotoxicity, as already explored in many studies.^25–27^ This fact has led to the stereoselective chemical synthesis of an enantiomeric mixture that contains 75% and 25%, respectively, of the S(−) and R­(+) BVC isomers (BVC_S75_, commercialized as Novabupi). Due to its lower affinity for sodium, potassium, and calcium channels than the commercial racemic mixture, BVC_S75_ exhibits reduced arrhythmogenesis and neurotoxicity, resulting in lower toxicity to the central nervous and cardiac systems^28,29^ and providing a greater safety margin.^17,30^

The effects of BVC on various melanoma cell lines have been reported, including reduced cell survival, disruption of cytoskeletal organization, impairment of energetic metabolism, and the inhibition of cell proliferation and pulmonary metastasis.^10,16,31^ In other tumor types, BVC induces apoptosis via caspase-dependent and caspase-independent pathways,^32,33^ the production of reactive oxygen species (ROS) through the activation of AMP-induced protein kinase (AMPK),^34^ and inhibition of cell proliferation and metastasis by suppressing PI3K/Akt and MAPK signaling pathways.^35^ However, to our knowledge, no *in vivo* investigation has been carried out for bupivacaine or its stereoisomers to confirm their antiproliferative effect in an entire organism.

A second generation of lipid-based DDS, the nanostructured lipid carriers (NLCs), emerged in the last decade of the 20th century.^36,37^ These nanoparticles exhibit an internal architecture composed of a blend of solid and liquid lipids stabilized by surfactants. The blend reduces the crystallinity of the matrix, increasing their upload capacities and preventing premature release during storage.^36–40^ NLCs are a valuable tool for modulating biodistribution, decreasing systemic toxicity, and maximizing the concentration of active pharmaceutical ingredients in the target tissue.^5,41,42^ Consequently, NLCs may be used to increase the efficacy of antineoplastics in cancer treatment while decreasing their toxic effects.^36,38,41^ In this regard, the encapsulation of LA in NLCs can enhance their antimetastatic and pro-apoptotic properties by prolonging their local action time, enhancing their antitumoral potential and reducing their systemic effects.

Moreover, NLCs can be prepared with functional excipients, such as natural lipids (waxes, essential oils), which add their intrinsic pharmaceutical properties to the formulation.^41–43^ In this context, we decided to use lavender essential oil (LO) as a functional excipient in an NLC to promote a synergistic effect with BVCS75. LO contains monoterpenes, such as linalyl acetate (28.6%), linalool (27.7%), eucalyptol, terpinen-4-ol (5.0%), caryophilene (3.9%), and lavandulyl acetate (3.7%). Therapeutic actions have been reported for these monoterpenes,^44–46^ such as sedative, antidepressant, antimicrobial, antifungal, antioxidant, and antineoplastic effects.^45–47^ Linalyl acetate, for instance, can inhibit melanogenesis in melanoma cells through oxidative stress by reducing tyrosine kinase activity through the regulation of JNK and ERK signaling pathways, promoting increased sensitivity to the cytotoxic action of chemotherapeutic agents in tumor cells.^47–49^ The activity of such LO components has also been reported in various human tumor cell lines in culture: neuroblastoma (SHSY5Y), breast adenocarcinoma (MCF-7), colorectal adenocarcinoma (Caco2), and lymphoblastic leukemia (CCRF-CEM).^50^

Several preclinical trials have investigated the effects of LA on metastasis control and tumor regression in animal models.^51–57^ However, the antitumor action of bupivacaine encapsulated in a delivery system within melanoma cells remains to be clarified. In light of this, we employed a primary melanoma induction model.^58^ Melanoma is the most lethal form of skin cancer, accounting for 73% of global deaths caused by cutaneous cancer.^59^ Its severity can be attributed to the tumor’s notable propensity for invasion, metastasis, heterogeneity, and therapeutic resistance, facilitating its aggressive behavior.^4^ The gold standard for treating primary melanoma involves wide excision, with margins determined by the tumor’s thickness,^4,60^ in which the use of LA is common.^61^

This work describes a potential therapeutic and innovative approach to improve LA efficacy in onco-anesthesia, utilizing BVC_S75_ encapsulated in an NLC composed of various (natural and synthetic) excipients in the treatment of melanoma. We present the results of local pharmacokinetic and efficacy studies to evaluate this nanometric system’s effectiveness.

## 2 Materials and Methods

### 2.1 Materials

Enantiomeric excess, 75:25 S(−):R­(+) mol % bupivacaine hydrochloride (BVC_S75_) was donated by Cristália Prod. Quim. Farm. Ltd. (Itapira, SP, Brazil). The natural products beeswax (BW) and lavender oil (LO) were purchased from Império das Essências (São Paulo, SP, Brazil) and Terra Flor Aromaterapia (Alto Paraíso de Goiás, GO, Brazil), respectively. Cetyl palmitate was purchased from Dhaymers Química Fina (Brazil), and Capryol 90 was donated by Gattefossé (France). Pluronic F68 (P68), Dulbecco’s modified Eagle’s medium (DMEM), fetal bovine serum, penicillin, and streptomycin were purchased from Sigma-Aldrich (St Louis, MO, USA). Deionized water (18 MΩ) was obtained with an Elga USF Maxima ultrapure water purifier (Elga Lab Water, High Wycombe, UK). American Type Culture Collection (ATCC, Manassas, VA, USA) provided murine melanoma (B16–F10 lineage). All other reagents were of analytical grade.

### 2.2 NLC Preparation

NLC formulations were prepared using the emulsification-ultrasonication method.^62^ BVC_S75_ was added to the lipid phase, composed of LO and BW, which was heated to 60 °C until complete solubilization. Simultaneously, an aqueous phase composed of P68 surfactant solution was heated to the same temperature, and both phases were mixed under high-speed agitation (11,000 rpm) for 3 min in an Ultra-Turrax homogenizer (IKA Werke, Staufen, Germany). The mixture was then tip-sonicated for 16 min in a Vibracell (Sonics & Mat. Inc., Danbury, CT, USA) sonicator at 60 W and 20 kHz, in alternating 30 s (on/off) cycles to avoid overheating the sample. Immediately afterward, the sample was cooled to room temperature in an ice bath to form nanoparticles (NLC-L-BVC); a blank formulation (NLC-L) without the anesthetic was also prepared.

For the sake of comparison, a second formulation (NLC-BVC) was prepared to clarify the effect of the excipient LO in the *in vivo* antineoplastic effect of the nanoparticles. Table 1 shows the composition of the NLC formulations.

**Table 1:** Composition of the Optimized (Natural and Synthetic) NLC Formulations Used in This Study

Lipid matrix	Formulation	Solid lipid	%	Liquid lipid	%	Surfactant	%	BVC_S75_ (%)
Natural	NLC-L	Beeswax	10.5	Lavender oil	4.5	Pluronic F-68	5	-
Natural	NLC-L-BVC	Beeswax	10.5	Lavender oil	4.5	Pluronic F-68	5	0.5
Synthetic	NLC-BVC	Cetyl palmitate	9.0	Capryol 90	4.0	Pluronic F-68	5	0.5

### 2.3 *In Vivo* Studies

#### 2.3.1 Animals

Female adult C57BL/6J mice aged 6–8 weeks (18–22 g) were obtained from the Multidisciplinary Center for Biological Research (CEMIB-UNICAMP). The experimental protocols were approved by the UNICAMP Institutional Animal Care and Use Committee (#5736-1/2021 and 5940-1/2022) that strictly follows the guidelines of the National Committee of Animal Experimentation (CONCEA, Brasília, DF, Brazil). The animals were housed at 25 ± 2 °C, 30–70% humidity, and under 12/12 h light/dark cycles, with food and water available *ad libitum*.

#### 2.3.2 Tumor Cell Inoculation

After the animals were intraperitoneally anesthetized with ketamine (100 mg/kg) and xylazine (10 mg/kg), they received aliquots (60 μL) of 1 × 10^6^ B16-F10 cells per mouse, subcutaneously into the right flank, to induce the tumor.^63^ The tumors were allowed to grow for 10 days (to 50–100 mm^3^) before treatment.

#### 2.3.3 Treatment Groups

On the 10th day after B16–F10 cell inoculation, when the tumor volume reached ∼100 mm^3^, the mice were randomly assigned into seven groups (*n* = 5 mice/group). Group 1 was the control (naive group) containing tumor-free animals. The animals in groups 2–7 had melanoma tumors and received the following intratumoral (IT) treatments: group 2 = 60 μL saline solution (negative control); group 3 = dacarbazine (positive control); group 4 = free BVC; group 5 = NLCs prepared with LO (NLC-L); group 6 = NLCs prepared with LO plus BVC_S75_ (NLC-L-BVC); and group 7 = LO-free NLCs plus BVC_S75_ (NLC-BVC).

#### 2.3.4 Pharmacokinetic Study (Local Microdialysis Inside the Tumor)

The pharmacokinetic evaluation of BVC_S75_ (free and encapsulated) in the melanoma model was performed by tissue microdialysis^64–66^ using CMA 20 probes (4 mm, CMA Microdialysis, Kista, Switzerland). Microdialysis was carried out on the animals between the 10th and 14th days after melanoma induction. Previously, the probes were calibrated *in vitro* to ensure that their relative recovery of BVC_S75_ by gain (dialysis) and by loss (retrodialysis) were similar.^67^ A BVC_S75_ concentration of 5 μg/mL in perfusion fluid consisting of 0.05 M phosphate buffer at pH 7.4 was used for probe calibration. A 1.5 μL/min flow rate was maintained using a PHD22/2000 infuser (Harvard Apparatus, MA, USA) with 1 mL syringes. A previously described HPLC bioanalytical study was employed for BVC_S75_ quantification in the microdialysate samples.^31^ The samples were injected directly into the HPLC system without further processing.

The procedure was carried out as shown in Figure S1. After the tumor reached a volume between 200–400 mm^3^, mice from the free BVC, NLC-L-BVC, and NLC-BVC treatment groups (*n* = 5 per group) were anesthetized with urethane (1,000 mg/kg) prior to sample collection. After anesthesia, the probes were inserted tangentially into the upper third portion of the tumor and left in place for 1 h to stabilize. Formulations containing 5 mg/mL BVC_S75_, in solution or encapsulated, were injected into the center of the tumor. The dialyzate was collected for unbound BVC_S75_ quantification by HPLC after 3 min and then at 30 min intervals. In two animals from each group, after the experiment, the buffer solution was replaced with 0.5% BVC_S75_, and the probe was stabilized for 1 h for subsequent collection of 3 × 30 min samples and calculation of *in vivo* retrodialysis (*recovery by loss*). The other three animals from each group were euthanized, and their tumors were dissected. The dissected tissue was subjected to acid extraction^68^ to determine the residual concentration of BVC_S75_ in the tumor.

PKanalix software (version 2021, Lixoft^©^) was employed to calculate the following pharmacokinetic parameters from the in situ BVC_S75_ concentration–time data: elimination rate constant (ke), tissue half-life (*t*_1/2_), area under the unbound concentration–time curve (AUC), and initial unbound tissue concentration (C_0_). Each parameter was statistically evaluated by two-way ANOVA (α = 0.05) followed by Tukey’s test using GraphPad Prism software, version 8 (California, USA).

NLC formulations were labeled with 0.01% rhodamine PE for subsequent fluorescence analysis of the tumor to confirm the permanence of the nanoparticles within it. Histological slides of the tumor tissue from euthanized animals were analyzed under an inverted microscope equipped with epi-fluorescence lighting and a digital camera (Leica DMI6000) for image capture.

#### 2.3.5 Antitumor Activity in Melanoma-Induced Mice

After tumor growth (Section 2.3.2), four sessions of each treatment were carried out (as described in Section 2.3.3), with a two-day interval between sessions. The animals were also observed daily for possible signs of adverse reactions (lethargy, inability to walk, weight loss). The animals’ body mass and feed were quantified weekly using an analytical balance (AND HM-202, EUA) with a digital scale. Every 2 days throughout the study, the dimensions (length and width, in mm) of the tumors were measured with a PD 200 (Vonder, Jundiaí, SP, Brazil) digital caliper (Figure 3A). Tumor volumes (mm^3^) were calculated according to eq 1 ^69^ Seven days after the end of the treatments, the animals were euthanized with ketamine (300 mg/kg) and xylazine (30 mg/kg) intraperitoneally.

The chemotherapy agent dacarbazine is a reference drug for the treatment of metastatic melanoma.^70,71^ It was used in this study as a positive control at a dosage of 80 mg/kg.^72^ As for BVC_S75_, we used a concentration (8 mg/kg) that took into consideration the anesthetic’s clinical doses.^73^ In the animals treated with the control formulation, i.e., NLC without bupivacaine (NLC-L), the injected formulations had the same particle concentration (3.1 × 10^13^ particles/mL) as those of the NLCs containing BVC_S75_ (NLC-L-BVC and NLC-BVC).

1 $$\mathrm{Tumor volume}=\frac{\mathrm{tumor length}\times \mathrm{tumor widt}h^{2}}{2}$$

#### 2.3.6 Toxicological Analysis

##### 2.3.6.1 Biochemical Analytes in Serum

Immediately after euthanasia, blood samples were taken via cardiac puncture of the animals in each treatment group (as described in Section 2.3.3) for the measurement of biochemical analytes in serum: alanine aminotransferase (ALT, IU/L), aspartate aminotransferase (AST, IU/L), creatine kinase-MB isoform (CK-MB, IU/L), and urea (mg/dL). ALT, AST, CK-MB, and urea were analyzed using the kinetic method. All measurements were carried out using the automated AU5800 Series Clinical Chemistry Analyzer (Beckman Coulter).

##### 2.3.6.2 Histopathological Analysis

After euthanasia and blood collection, the mice were placed on a surgical field, and, using a scalpel, a straight frontal incision was made for the excision of the spleen, liver, lungs, kidneys, and tumor/skin. The collected organs and the tumor were then placed in containers with 10% formaldehyde solution and phosphate buffer (pH 7.4). Morphological analyses were carried out according to the standard protocol established in the literature.^74^ The slices were stained with hematoxylin/eosin (HE), analyzed/photographed using a Leica optical microscope (Leica Microsystems, Switzerland) at 5 × and 10 × magnifications, and processed with Leica 4.2.0 software (Leica Microsystems, Switzerland).

Clark’s classification was used to assess the degree of tumor invasion in the animal’s epidermis as follows: I) the cancer is restricted to the epidermis, II) there is invasion of the papillary dermis, III) the tumor fills the entire papillary dermis without invading the reticular dermis, IV) there is invasion of the reticular dermis, and V) there is invasion of the hypodermis.^5,75,76^ The prognosis for melanoma therapy is related to the depth and thickness of tumor cell invasion into the epidermis.^76,77^ The histological criteria considered to be a response to the tested treatments were mainly necrosis,^78^ reduction in the size of the neoplastic region,^79^ the presence of inflammatory infiltrate,^80,81^ and stromal fibroplasia.^82,83^

##### 2.3.6.3 NMR-Metabolomics

To access the metabolic profile of the animals’ livers, after euthanasia, the tissue was dissected, frozen in liquid nitrogen, and conserved at −80 °C. Tissue homogenization and metabolite extraction were carried out in 50 mg samples of frozen liver tissues resuspended in 1 mL of cold phosphate buffer (100 mM, pH 7.4) and agitated in a Potter–Elvehjem homogenizer. Subsequently, 1 mL of the homogenate was mixed with methanol:chloroform:water (1:1:0.8, v/v) for metabolite extraction. Finally, the samples were centrifugated at 1000× g for 10 min, and the aqueous phase was collected, dried in a vacuum concentrator, and conserved at −80 °C until NMR analysis. For that, the dry samples were resuspended in 600 μL of D_2_O-phosphate buffer (0.1 M, pH 7.4) and 0.5 mM of trimethylsilylpropionate (TMSP-d4 signal). The samples were transferred to 5 mm NMR tubes for spectra acquisition in a Varian Inova spectrometer (Agilent Technologies Inc., Santa Clara, CA, USA) equipped with a triple-resonance cold probe and operating at a 1H resonance frequency of 600 MHz.^1^ H NMR spectra acquisition was performed with 256 scans collected with 32 K data points over a spectral width of 8,000 Hz. 2D NMR ^1^H–^1^H-TOCSY spectra were acquired using a spectral width of 8,000 Hz and 128 increments with 56 transients of 2k complex points for each free induction decay. 2D NMR ^1^H–^13^C-HSQC spectra were recorded with a spectral width of 8,000 Hz × 25,133 Hz and 128 increments with 60 transients of 2k complex points. In both 2D NMR spectra and 1H-NMR spectra, a 1.5-s relaxation delay was incorporated between scans with a continual water presaturation radiofrequency (RF) field to eliminate the residual water signal. Spectra were converted and processed with TopSpin 4.0.3 (Bruker BioSpin, Rheinstetten, Germany), cosine multiplication (ssb 2), zero-filling to 64 k data points, manual phasing, baseline correction, and calibration to TSP-d4/TMS signals (δ 0 ppm). The signal assignment was based on matching 1D spectral information to reference spectra available in Chenomx 9.0 (Edmonton, AB, Canada) and the human metabolic database (HMDB) (www.hmdb.ca). In instances where confirmation was required, the 2D 1H-1H-TOCSY/^1^H–^13^C-HSQC spectra were employed to validate the identity of specific metabolites.

Spectral integration and total area normalization of selected signals were carried out in Amix-Viewer 3.9.15 (Bruker Biospin, Rheinstetten, Germany) to provide a quantitative measurement of metabolic variations. Afterward, the metabolome data were analyzed using MetaboAnalyst 6.0 (https://metaboanalyst.ca/), in which data was normalized by median, and autoscaling was used. After, multivariate analysis (principal component analysis (PCA) and sparse partial least-squares discriminant analysis (sPLS-DA)) was employed along with univariate analysis (a heatmap was constructed with significant metabolites calculated by ANOVA (*p* < 0.05), using the Pearson distance measure and Ward’s clustering algorithm).

#### 2.3.7 Animal Survival

The survival probability of the animals in each treatment group (as described in Section 2.3.3) was calculated by the Kaplan–Meier method.^84^ The nonparametric log-rank test was used to compare the curves between the groups. Statistical data was generated with GraphPad Prism version 8.0.1 (GraphPad Software Inc., La Jolla, California, USA); *p* < 0.05 was considered significant. Humanitarian intervention was carried out when the tumor exceeds 10 mm diameter or in cases of tumor ulceration, a 20% loss of the animal’s body mass, loss of appetite, changes in mobility, and physiological behavior. The approval certificates for the experimental protocols (#5736-1/2021 and #5940-1/2022) are provided in Section 5 of the Supporting Information.

### 2.4 Statistical Analysis

The results were presented as mean ± standard deviation (SD). Statistical analyses were conducted using GraphPad Prism, version 8.0.1. When comparing multiple groups, a two-way analysis of variance (ANOVA) was performed to assess whether there were significant differences among the tests conducted, followed by Tukey’s posthoc test, with *p* < 0.05 considered significant.

## 3 Results and Discussion

### 3.1 Physicochemical Characterization of the Prepared NLC Formulations

The formulations in this study were developed and optimized by experimental design (DoE) and characterized by various techniques—DLS, NTA, DSC, XRD, and TEM—regarding their BVC_S75_ *in vitro* release kinetics and cytotoxicity.^42,85^ As described in the Methods section (Table 1), an NLC formulation containing a synthetic liquid lipid but no lavender oil (NLC-BVC) was used for comparison with NLC-L-BVC to investigate whether lavender oil could enhance bupivacaine’s effect in melanoma treatment. Natural lipids, such as lavender oil, are highly biocompatible and easily metabolized, reducing the risk of long-term accumulation and adverse reactions. However, their variable composition may affect formulation consistency and reproducibility.^86^ Conversely, synthetic lipids provide a consistent chemical composition, greater stability, and lower cost but may be toxic and are not biodegradable, potentially leading to accumulation in the body.^87^ Table 2 summarizes the physicochemical properties of both NLC formulations, providing insights into their potential advantages and limitations for therapeutic use.

**Table 2:** Physicochemical characterization—Size, Polydispersity Index (PDI), Zeta Potential (ZP), Nanoparticle Concentration (NC), and BVC_S75_ Encapsulation Efficiency (%ee)—of the Formulations with Bupivacaine (NLC-L-BVC and NLC-BVC) and with Lavender Oil Alone (NLC-L)

Formulation	Size (nm)	PDI	ZP (mV)	NC (x 10^13^/mL)	%EE
**NLC-L**	195.4 ± 1.8	0.138 ± 0.03	-26.8 ± 0.8	3.1 ± 0.2	-
**NLC-L-BVC**	200.5 ± 2.4	0.140 ± 0.02	-34.0 ± 0.6	3.8 ± 0.3	89.0 ± 2.3
**NLC-BVC** ^a^	165.9 ± 1.5	0.123 ± 0.05	-37.0 ± 1.1	8.8 ± 0.1	91.0 ± 3.4

a See ref 85.

The data in Table 2 reveal nanoparticles of ideal size, PDI, ZP (homogeneous and not prone to fusion), and concentration for infiltrative application in tumor tissue.^88–90^ Both of the BVC_S75_-containing formulations exhibited high encapsulation efficiency.

### 3.2 Pharmacokinetic Study (Local Microdialysis in the Tumor)

Surgery on primary tumors associated with anesthesia has been an essential part of cancer therapy.^6,14,91^ In order to assess the pharmacokinetic parameters of BVC_S75_ (free and NLC-encapsulated) in melanoma tumors, we used the local microdialysis (directly in the tumor) technique, which allows the unbound drug concentration to be harvested over time from the tumor’s interstitial space fluid through the probe’s semipermeable membrane, perfused with an isosmotic solution.^67,92^

The *in vitro* calibration of the microdialysis probes resulted in BVC_S75_ relative recovery of 22.0 ± 1.0% by gain (dialysis), similar to the recovery of 23.0 ± 0.8% by loss (retrodialysis), indicating that the drug does not bond to the microdialysis system tubing and confirming that retrodialysis can be used to calibrate the probes *in vivo*. The BVC_S75_ average *in vivo* relative recovery was 14.7 ± 0.9%. This average value was used to back-calculate the real unbound BVC_S75_ concentrations inside the tumor.

BVC_S75_ concentration inside the tumor tissue was plotted as a function of sample collection time, as shown in Figure 1.

**Figure 1:**
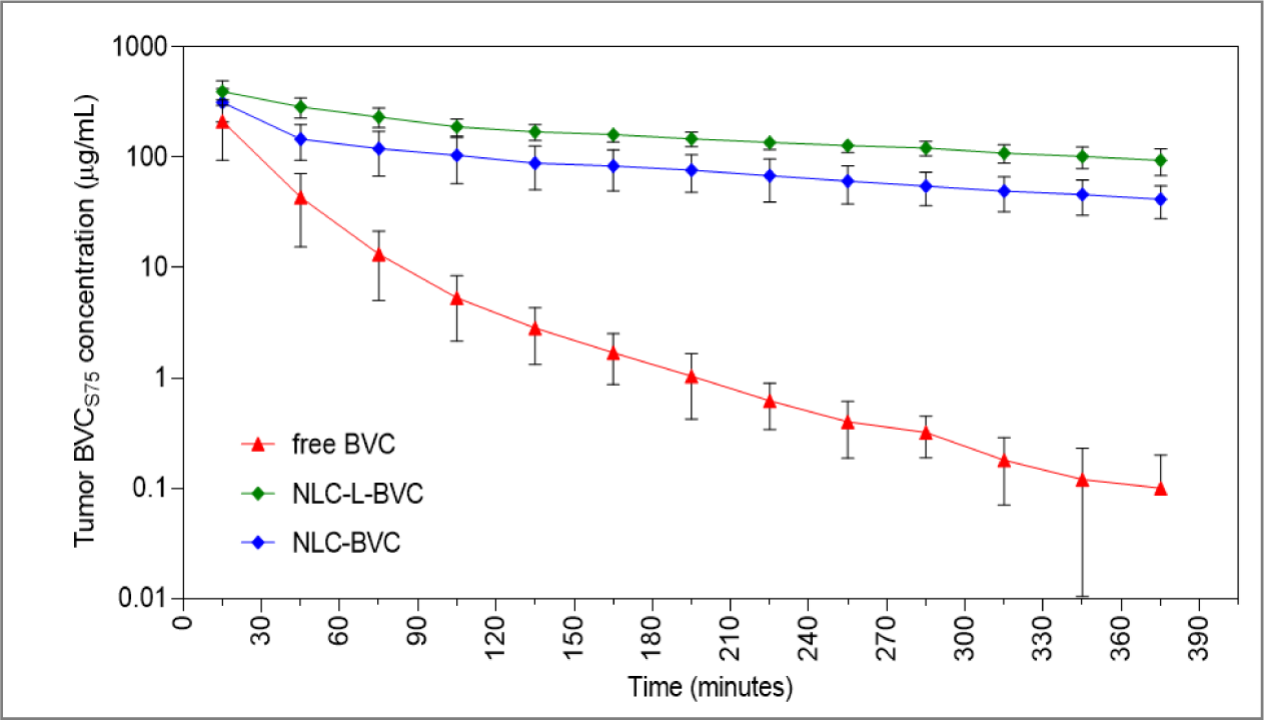
Local unbound concentration inside the tumor as a function of time. Data represent mean ± SD (*n* = 5). Free BVC = unbound solution of 0.5% bupivacaine S75:R25; NLC-L-BVC = nanostructured lipid carrier with lavender oil and 0.5% of BVC_S75_; NLC-BVC nanostructured lipid carrier with 0.5% of BVC_S75_; and no lavender oil.

As expected, the unbound concentrations of BVC_S75_ inside the tumor decreased with time, but the decrease was significantly faster in the animals treated with free BVC_S75_ than in the groups treated with encapsulated BVC_S75_. Therefore, nanoencapsulation in NLC-L-BVC or NLC-BVC promoted a sustained release of BVC_S75_, in agreement with previous results from *in vitro* release kinetics,^42,85^ increasing the LA concentration in the tumor tissue.

The pharmacokinetic parameters determined from the unbound BVC_S75_ concentration inside the tumor (Table 3) show how drug encapsulation into the nanoparticles affected the anesthetic’s pharmacokinetics.

**Table 3:** Pharmacokinetic Parameters Calculated from the BVC_S75_ Unbound Concentration–Time Profiles inside the Tumor Tissue

Parameters	free BVC	NLC-L-BVC	NLC-BVC
ke (h^–1^)	0.91 ± 0.19	0.16 ± 0.08^a^	0.18 ± 0.10^a^
*t*_1/2_ (h)	0.80 ± 0.17	5.8 ± 3.60^b^	4.9 ± 2.70^b^
AUC_0‑∞_ (μg·h/mL)	178 ± 96	2005 ± 712^a^	1243 ± 230^c^
C_0_ (μg/mL)	509.1 ± 315.2	464.8 ± 127.7	463.3 ± 159.1

a *p* < 0.0001 in comparison to free BVC_S75_.

b *p* < 0.05.

c *p* < 0.01.

The release profile of BVC_S75_ encapsulated in NLC was like that determined by previous *in vitro* release assays.^42,85^ The unencapsulated drug fraction led to rapid release (“*burst effect*”) observed as a high concentration at time zero (C_0_). BVC_S75_ fractions encapsulated in NLCs’ lipid cores also caused high initial concentrations but then promoted a sustained release observed until the end of the experiment.

Although it cannot be visualized in Figure 1 due to the log scale (Y-axis), high variability was observed for the nanoformulations’ concentration–time profiles. Therefore, no statistically significant (*p* < 0.05) differences were detected between the nanoformulations’ pharmacokinetic metrics. A comparison between the nanoformulations and the free drug showed that free BVC_S75_’s elimination rate constant in the tumor tissue, 0.91 ± 0.19 h^–1^, was around 5- to 6-fold faster than that estimated for the NLC-L-BVC and NLC-BVC formulations (Table 3). The half-life time increased from ∼1 h (free BVC) to 5–6 h when the drug was encapsulated in either type of NLC, with a significant difference (*p* < 0.05) between the free BVC and NLC-L-BVC groups. Accordingly, the AUC was higher in the groups treated with NLC-L-BVC (2005 ± 712 μg·h/mL, *p* < 0.001) and NLC-BVC (914 ± 204 μg·h/mL, *p* < 0.05) than those treated with free BVC (178 ± 96 μg·h/mL), proving that the nanoformulations promote increased exposure of the tumor to the anesthetic.

At the end of the experiment (after 6 h of collection), the animals were euthanized, and the BVC_S75_ concentration in the tumors was quantified by HPLC. BVC_S75_ concentrations were around four times higher in the animals treated with the nanoformulations than in those treated with the free drug (*p* < 0.05), as shown in Figure 2A. Subsequently, the NLC formulations were labeled with rhodamine and applied to the tumor to determine the presence of the nanoparticles in the tumor tissue. The histological slides of the tumor tissue from euthanized animals were analyzed under a fluorescence microscope. Figure 2B depicts the tumor of an animal treated with free BVC, which has no red fluorescence. The presence of rhodamine in the tumor tissue (shown by the red color in Figure 2C,D) is an indication that nanoparticles are still in the tumor after the experiment and that the concentration of BVC_S75_ is higher in tumors treated with NLC formulations, revealing that the NLC prolongs drug release at the site of action, protecting BVC_S75_ from normal clearance and favoring its interaction with tumor cells.^41,93^

**Figure 2:**
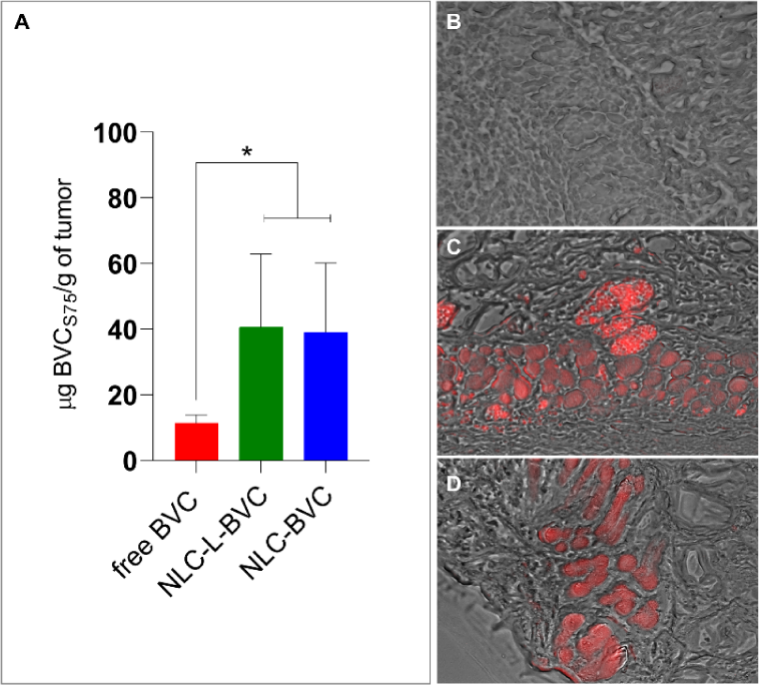
(A) Residual posteuthanasia intratumoral BVC_S75_ concentration after 8 h. Fluorescence microscopy images of tumors treated with free BVC (B), NLC-L-BVC (C), or NLC-BVC (D). The NLC formulations were labeled with red fluorescent rhodamine-PE. Statistical analysis carried out by unpaired Student’s t-test; * *p* < 0.05.

### 3.3 In Vivo Antitumor Activity in Melanoma-Induced Mice

#### 3.3.1 Effect of Treatments on the Primary Tumor

The intradermal implant of B16–F10 tumor cells in C57BL/6J mice is a common studies model, as it produces aggressively growing tumors with invasive and metastatic profiles, given the tumor’s vertiginous evolution.^58^ In this tumor induction model, six different treatments (see section 2.3.3) were administered intratumorally to determine their effect on the primary tumor. The evolution of the primary tumors and their histopathological features were analyzed both during treatment and post-treatment.

##### 3.3.1.1 Evolution of Primary Tumor During Treatment

The efficacy of the treatments on the primary tumor was evaluated considering the tumor volume (TV) during the four treatment sessions (16 days) and in the post-treatment as shown in Figure 3B. The group of animals treated with saline (negative control) showed rapid and exponential tumor growth. At day 16, treatment with dacarbazine (positive control) inhibited tumor volume by 30% compared to the negative control (*p* < 0.05). A small reduction of tumor volume (17%) was registered in the animals treated with free BVC, while treatments with NLC-L-BVC and NLC-BVC resulted in substantial TV reduction of 70% and 72%, respectively, compared to the negative control (*p* < 0.0001), as detailed in Figure S2B. The NLC formulation with lavender oil alone (NLC-L) reduced the tumor growth by 67% in comparison to the negative control (*p* < 0.0001). These data demonstrated that all NLC formulations tested reduced tumor growth.

**Figure 3:**
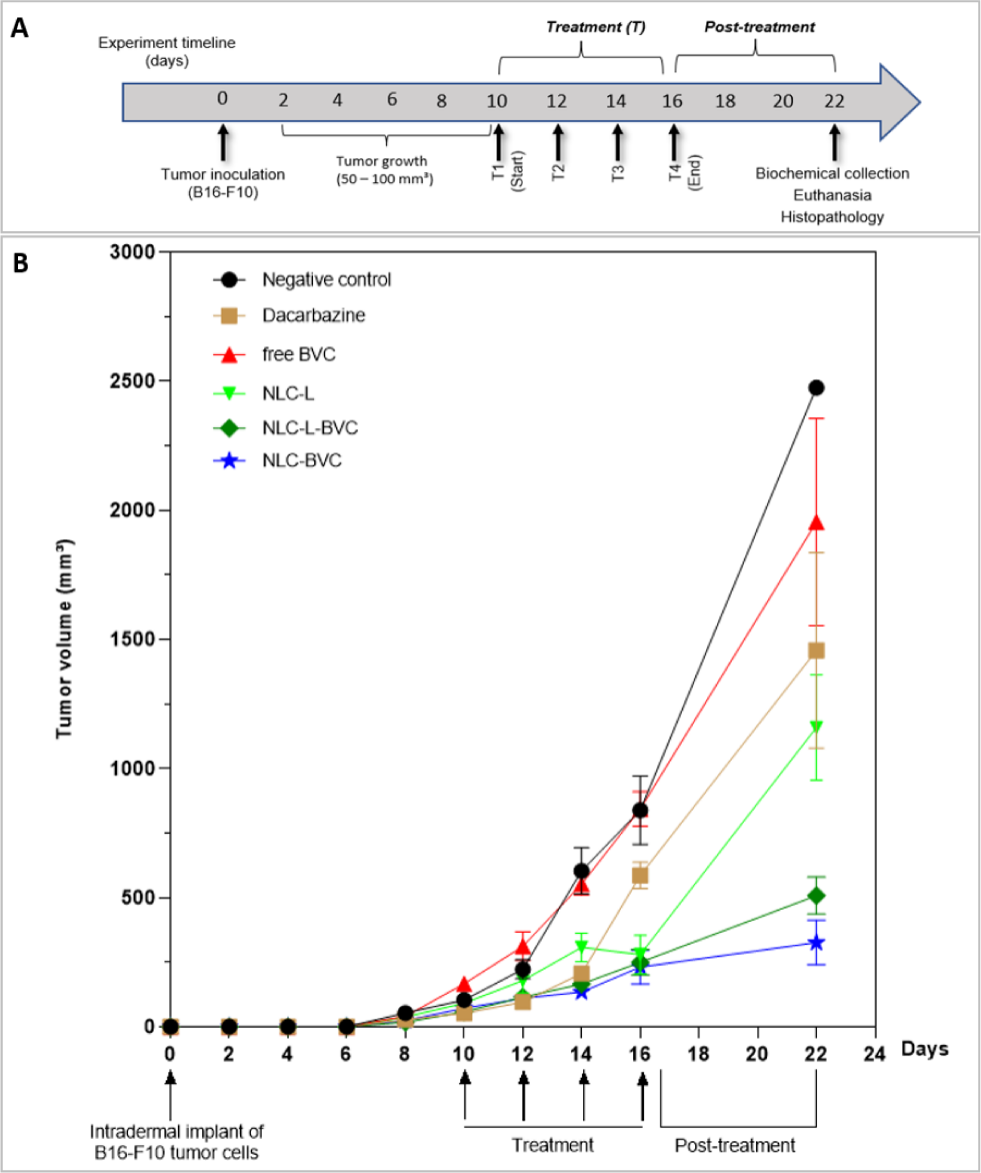
(A) Timeline of the experimental design: T1, T2, T3, and T4 = treatments. (B) Evolution of the primary tumor during and after treatment of animals with negative control = 0.9% NaCl, positive control = dacarbazine, free BVC (0.5%), lavender oil formulation (NLC-L), or the formulations containing 0.5% BVCS75 (NLC-L-BVC and NLC-BVC).

##### 3.3.1.2 Evolution of the Primary Tumor after Treatment

We analyzed the post-treatment effect on the growth of the animals’ primary tumors. Figure 4A shows the respective tumor volumes of the animals in each treatment group during the one-week period after the end of treatment (day 22). Figure 4B is a graphical representation of the size of the tumors excised at the end of this period.

**Figure 4:**
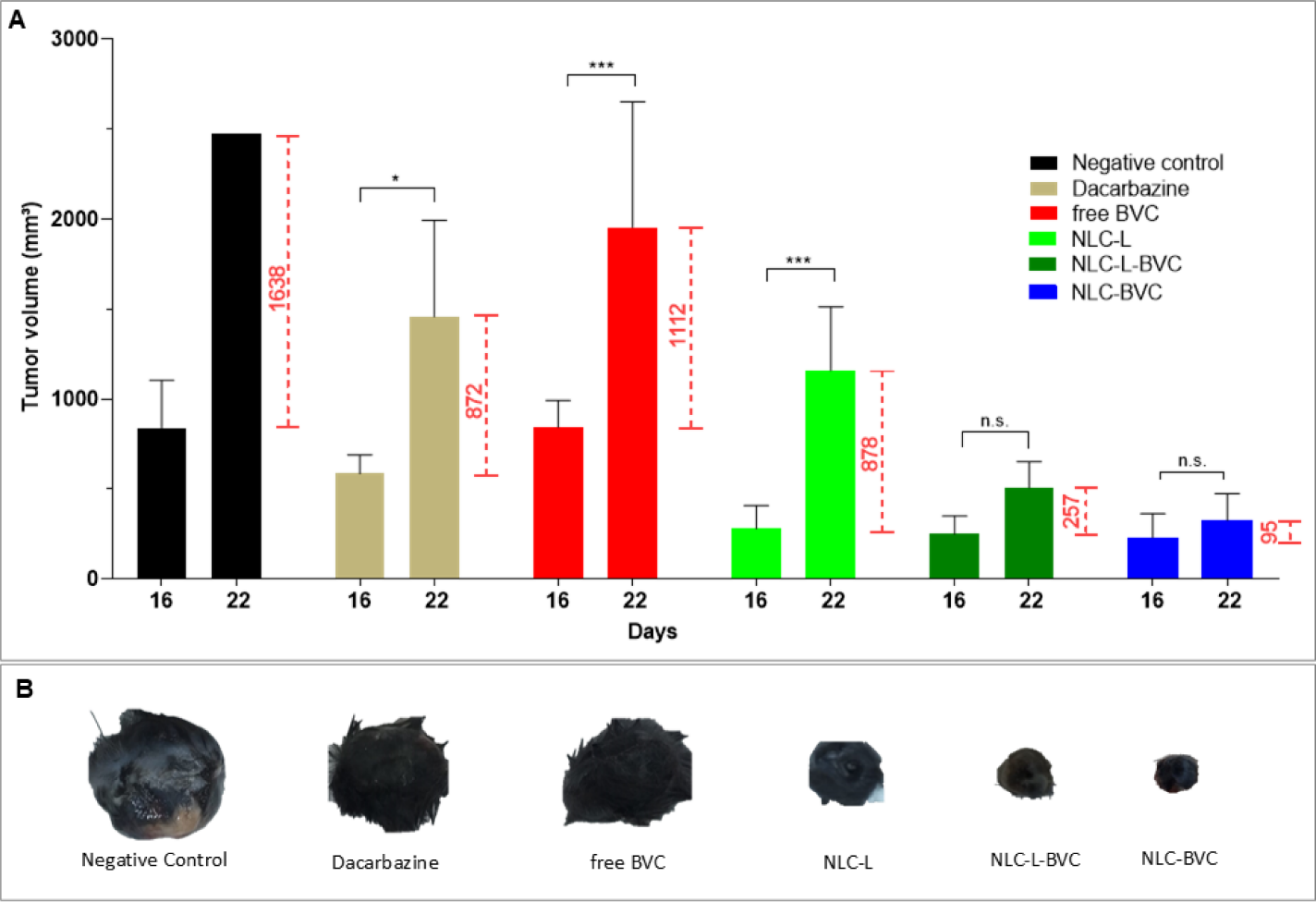
Analysis of post-treatment tumor growth. (A) Comparison of tumor volume at day 16 (last day of treatment) and 1 week after (day 22) the end of treatment with negative control (0.9% NaCl), positive control (dacarbazine), free BVC (0.5%), lavender oil formulation (NLC-L), or formulations containing 0.5% BVC_S75_: NLC-L-BVC and NLC-BVC. (B) Representative examples of tumors from each treatment, excised at day 22 (same scale). Statistical analysis: one-way ANOVA plus posthoc Tukey. * *p* < 0.05; *** *p* < 0.001; n.s. = nonsignificant. No statistical analysis was applied to the negative control group because only one animal in this group survived until day 22.

Figure 4A,B shows that intensive tumor growth was observed in the negative control group on day 22 (1638 mm^3^ of growth). Tumor growth was significantly reduced by treatments with NLC-L-BVC and NLC-BVC (257 mm^3^ and 95 mm^3^ of growth, respectively), which were more effective than treatment with dacarbazine or free BVC (872 mm^3^ [*p* < 0.05] and 1112 mm^3^ [*p* < 0.001] of growth, respectively). Among the treatments, formulations with BVC_S75_ encapsulated in NLC resulted in the least tumor growth, with no significant differences between the treatment and post-treatment periods. Conversely, the antitumor effect of the lavender oil nanoparticle (NLC-L) decreased in the post-treatment period; the tumor volume increased to 878 mm^3^, a significant difference from day 16 (*p* < 0.001), similar to what was observed with dacarbazine.

There was a significant reduction in tumor growth in the animals treated with NLC-L-BVC and NLC-BVC compared to the other treatments during the treatment and post-treatment period. Considering the pharmacokinetic findings, these results could be due to the prolonged release of BVC_S75_ promoted by the nanoparticles that not only allowed the anesthetic to remain in the tumor tissue for longer but also promoted a greater concentration of the drug at the site of action, improving the action of BVC_S75_. Moreover, as proposed by Nguyen et al., NLCs could modulate the microenvironment of solid tumors, enhancing drug efficacy and possibly blocking their cellular resistance mechanism,^90,94^ which may have contributed to the superior performance observed.

##### 3.3.1.3 Histological Analysis of the Primary Tumor

Histopathological images of the animals’ tumor groups are shown in Figure 5; the complete histopathological analysis is described in Section 3 of the Supporting Information (Figure S3). As expected, the animals in the naive group had normal skin with well-defined layers. The animals in the negative control group (untreated) showed an aggressive tumor profile characterized by extensive areas of necrosis and invasion of the underlying muscle and fat tissue, classified as Clark V (see “Methods”). Tumors in animals treated with dacarbazine and free BVC displayed a Clark IV level, as did the tumors of animals treated with NLC-L-BVC and NLC-BVC. Those treated with the NLC-L formulation displayed a Clark V level. Thus, the histopathological analysis showed that all the treatments (except NLC-L) decreased the degree of tumor invasion; BVC_S75_ encapsulation did not bring about a better result for this parameter. Additionally, tumors treated with the NLC-L formulation presented an aggressive profile similar to untreated tumors, which could result in a worse post-treatment prognosis.

**Figure 5:**
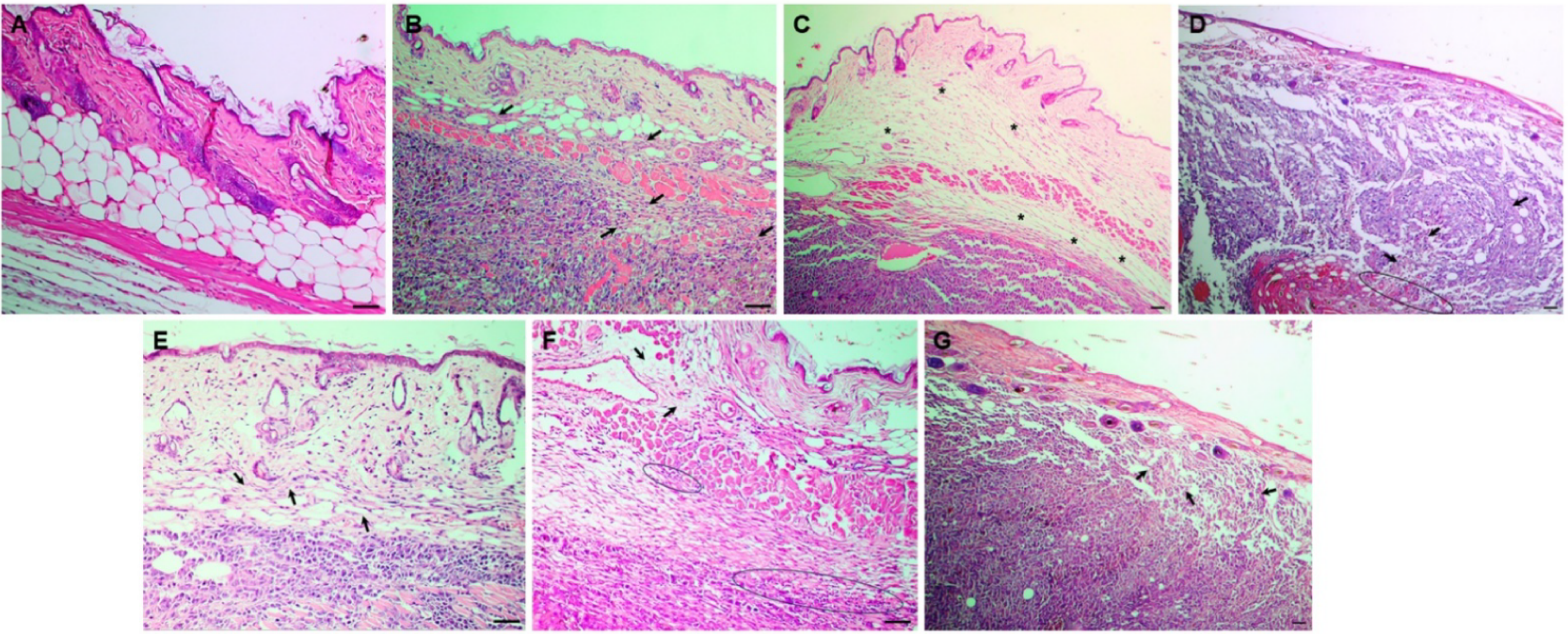
Histopathological sections of excised tumors (H&E staining): naive (A), negative control (B), dacarbazine (C), free BVC (D), NLC-L-BVC (E), NLC-BVC (F), and NLC-L (G). Scale bar = 100 μm, magnification: 5× and 10×. The black arrows point to areas of necrosis, asterisks represent areas of edema, and circled areas show inflammatory infiltrate.

Despite the recognized antitumor properties of lavender oil,^45,95^ its *in vivo* efficacy did not align with expectations and contrasts with the *in vitro* results reported in a previous study^42^ since an aggressive tumor profile was seen after NLC-L treatment (Figure 5G). Moreover, the combination of LO and BVC_S75_ in the same nanoparticle did not produce a synergistic effect for the local treatment of melanoma. This is likely attributable to the complexity of the tumor microenvironment and other pharmacological aspects affecting the response to the oil and the combined therapy. Unfortunately, it is not uncommon in cancer research for promising *in vitro* results to fail to translate into *in vivo* outcomes.^96,97^

In the next step, the systemic effects of the treatments were evaluated.

### 3.4 Systemic Evaluation of the Treatments’ Effectiveness and Toxicological Profiles

Clinical, biochemical, and histopathological parameters were assessed to evaluate the effectiveness and toxicological profile of the treatments. Body weight and food intake are key clinical parameters for assessing possible adverse effects caused by a neoplasm, treatments, or both.^98,99^ The biochemical analytes ALT, AST, CK-MB, and urea were assessed for possible treatment-related changes in the animals’ metabolic processes,^100^ which also provided the NMR metabolomic analysis of the liver.^101^ Finally, histopathological analyses of the organs were carried out to evaluate the diagnostic perspective and the effects of the treatments on the tissues.^102^

#### 3.4.1 Weight and Ingestion of Food

The weight and feed consumption of the animals were analyzed; the results are shown in Figure 6. There was a significant decrease in the weight of the animals in the negative control (*p* < 0.0001), dacarbazine, and NLC-L (*p* < 0.001) groups, in which the tumor growth was greater than 870 mm^3^, compared to the naive group (Figure 4A). No significant decrease was observed in the body mass of the animals treated with free BVC. Similar results were also seen in the animals treated with both kinds of NLC containing BVC_S75_ (Figure 6A), reinforcing the possible role of BVC_S75_ in improving the prognosis in the case of neoplasms.^6,14^

**Figure 6:**
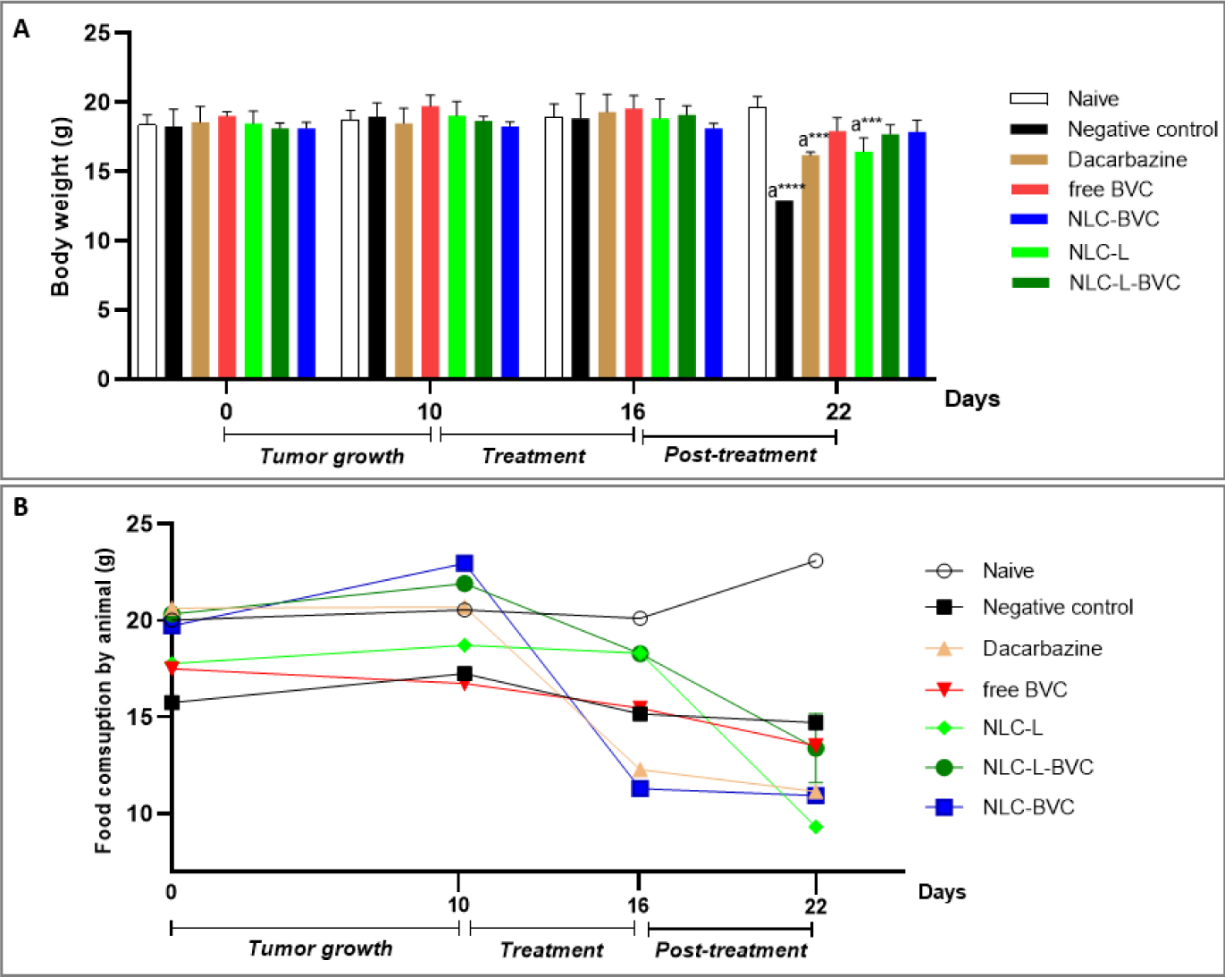
Weight analysis and feed consumption during the experiment. Average weight (A) and feed consumption (B) by animals without treatment (naive) and treatment with negative control (0.9% NaCl), positive control (dacarbazine), free BVC (0.5%), lavender oil formulation (NLC-L), or formulations containing 0.5% BVC_S75_: NLC-L-BVC and NLC-BVC. Statistical analysis: two-way ANOVA plus posthoc Tukey. a = in comparison to the naive group. *** *p* < 0.001; **** *p* < 0.0001.

Average feed consumption (Figure 6B) revealed no significant differences in treated animals in relation to the naive group during the experiment. A reduction in feed consumption was only observed in the post-treatment period in the negative control, dacarbazine, and NLC-L groups, which explains the loss of body mass (Figure 6A) and lower survival rate (Figure 9B) observed in these animals.

#### 3.4.2 Biochemical Profile

Biochemical analyses of alanine transaminase (ALT), aspartate aminotransferase (AST), creatine kinase MB (CK-MB), and urea were performed on the serum of treated groups of animals (Figure 7). ALT and AST are the most used markers for the clinical diagnosis of liver damage,^103,104^ while elevations in serum urea levels can indicate renal overload or nephrotoxicity.^105–107^ CK-MB is a key biomarker in diagnosing cardiac damage, and as recently reported, it can be altered in colorectal, lung, and hepatocellular cancers.^108,109^

**Figure 7:**
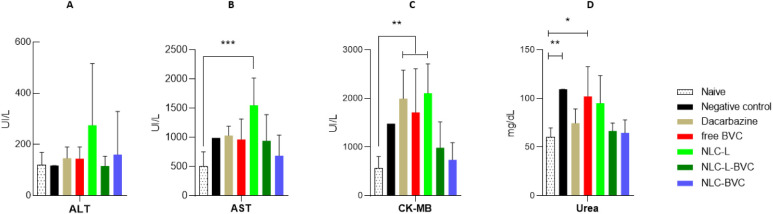
Biochemical parameters: serum ALT, AST, CK-MB activity, and urea levels in the post-treatment period of animals treated with 0.9% NaCl, dacarbazine, free BVC, NLC-L, NLC-L-BVC, or NLC-BVC. ALT (A), AST (B), CK-MB (C), and urea (D). Statistical analysis: one-way ANOVA plus posthoc Tukey. *p < 0.05; **p < 0.01; ***p < 0.001.

Among the markers of liver damage, no significant changes were observed at the tumor level (untreated group) compared to the healthy group. In terms of treatment toxicity, there was an increase in AST levels only in the NLC-L treatment group (Figure 7B), which may indicate a liver response to the metabolization of LO.

Regarding the CK-MB levels (Figure 7C), it was observed that free BVC, dacarbazine, and NLC-L treatments promoted changes in relation to the naive group (*p* < 0.05). Concerning the animals treated with dacarbazine, the increase in CK-MB corroborates reports in the literature on the cardiotoxicity of this antineoplastic drug.^110,111^ The increase in CK-MB levels induced by free BVC was not observed in the groups treated with encapsulated BVC_S75_ (NLC-L-BVC and NLC-BVC), for which CK-MB levels were comparable to those of the naive group. This indicates that encapsulation reduces the cardiotoxicity of free BVC.

As for serum urea levels (Figure 7D), the primary tumor increased the concentration of this metabolite. Among the treatments, only free BVC increased urea levels compared to the naive group (*p* < 0.05). This increase was not observed in the groups treated with encapsulated BVC_S75_, revealing that the prolonged drug release promoted by the nanoparticles (Figure 1) promotes greater effectiveness in treating the primary tumor at a systemic level.

Finally, the tissue-to-body weight coefficients (for the liver, heart, lungs, kidney, and spleen), which relate the weight of specific tissues to the total body weight of the animal, were calculated as the ratio of the tissue’s wet weight (mg) to body weight (g) in order to assess potential anatomopathological changes and toxicity^112^ (Figure S4). The data indicated that the heart, lungs, and kidneys of animals with tumors showed no significant changes compared to the naive group. However, in the liver, tumor induction led to an increase in its weight relative to the animal’s body weight (tissue hypertrophy), which was mitigated by the treatments, except for dacarbazine, corroborating the direct effectiveness observed in the primary tumor. There was an increase in the weight of the spleens of all tumor-induced animals, which treatments with an NLC containing bupivacaine did not significantly impact.

#### 3.4.3 NMR Metabolomics of the Liver

NMR metabolomic analysis is a technique used in cancer and drug toxicology studies to identify the changes caused by these agents in normal and tumor tissues.^101^ The liver was chosen for analysis because it is a common site of metastasis in this work’s model^113^ and due to its importance in various biochemical processes besides the metabolization of xenobiotics.^114,115^ Two aspects were considered: first, whether the tumor induced metabolic changes in the liver (and how treatments influenced these changes) and, second, whether treatments themselves caused metabolic disturbances that could indicate toxic effects.

The hydrophilic extracts from the liver samples were analyzed using ^1^H NMR spectroscopy, which allowed for the identification of 34 distinct metabolites, as illustrated in the reference spectrum provided in Figure S5. Subsequently, both multivariate and univariate analyses were performed on the liver metabolomic data, and the detailed results of these analyses are presented in Figure 8. PCA analysis (Figure 8A) revealed a clear separation between the naive and negative control (nontreated) groups, indicating cancer-induced metabolic alterations in this tissue. When analyzing the sPLDA loadings (Figure 8B), which show the main metabolites responsible for separating the groups, the metabolite hypoxanthine was shown to be the most important variable (>0.4) and was increased in the negative control group (Figure 8C). It has been reported in the literature that an increase in hypoxanthine is commonly found in tumor cells; consequently, it is considered a tumor biomarker.^116^ Regarding the treatments, the NLC-L, NLC-L-BVC, and NLC-BVC nanoformulations promoted a decrease in hypoxanthine (Figure 8D), indicating their effectiveness. These data agree with the observed reduction in tumor growth (Figure 4B).

**Figure 8:**
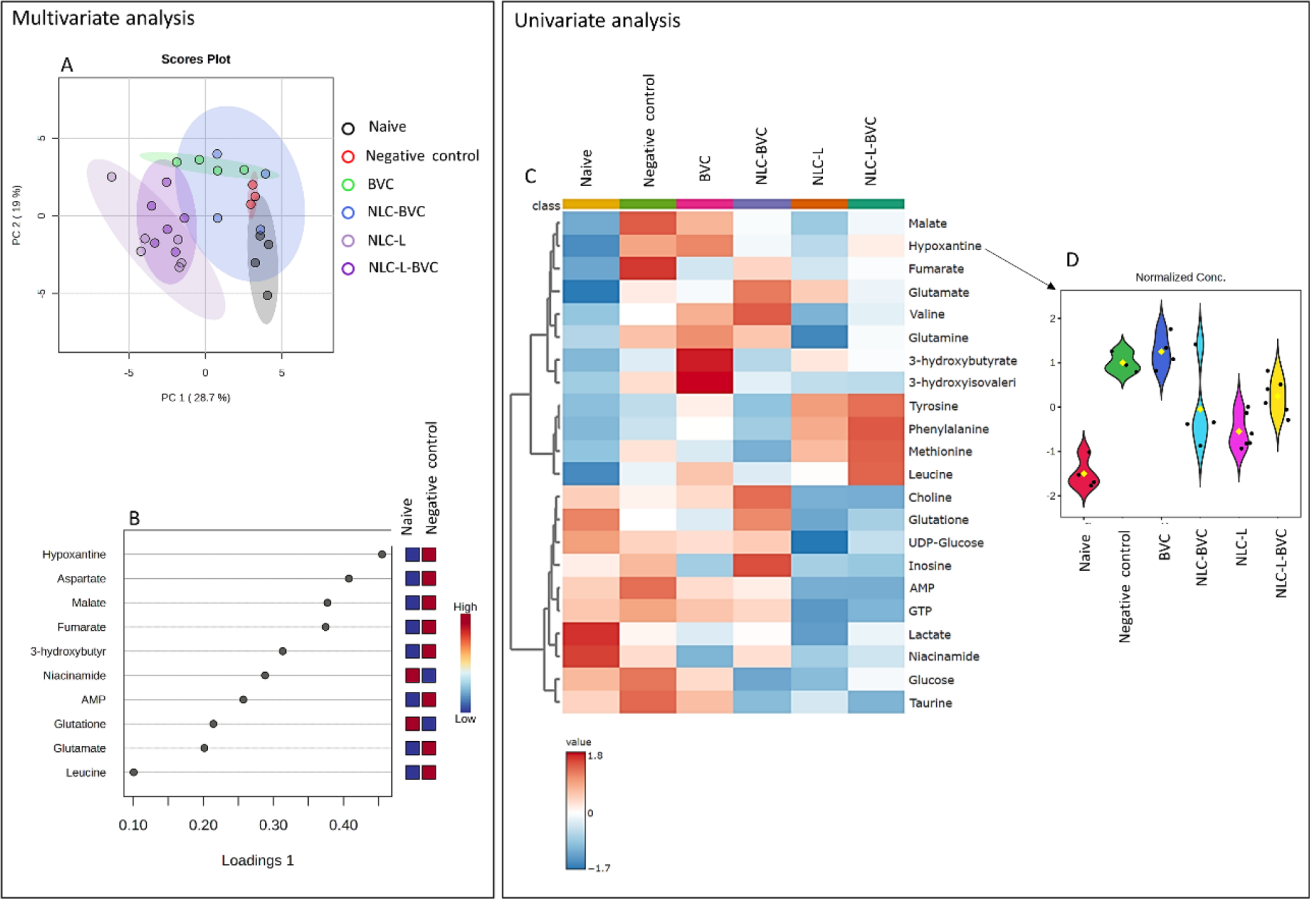
Results of metabolic profiling of the liver. Multivariate analysis (left): (A) principal component analysis (PCA) and (B) sparse partial least-squares discriminant analysis (sPLS-DA). Univariate analysis (right): (C) heatmap of significant metabolites (ANOVA, *p* < 0.05) and (D) violin plot for the metabolite hypoxanthine.

On the other hand, there was a clear separation in the PCA results involving groups of treatments that contained LO. The heatmap in Figure 8C reveals that the amino acids tyrosine, phenylalanine, methionine, and leucine are increased in relation to the naive and negative control groups. Additionally, the metabolites choline, glutathione, UDP-glucose, AMP, GTP, lactate, niacinamide, glucose, and taurine were diminished. This could indicate the liver’s response to the metabolization of LO, as also observed in the biochemical profile, with an increase in the serum concentrations of AST (Figure 7B). Even though there are no reports in the literature on the toxicity of this essential oil to the liver, these results indicate the requirement of further studies to understand the cause of these alterations and to determine whether they are a toxic response to LO, even when encapsulated in nanoparticles.

#### 3.4.4 Histopathologic Analysis of Organs (Spleen, Liver, Lungs, and Kidneys)

Histopathology analysis of the organs is shown in Section 4 of the Supporting Information (Figure S6). In summary, the liver, kidneys, and spleen revealed normal histological characteristics in all groups. The pulmonary evaluation revealed that the naive, negative control, NLC-L, and NLC-BVC groups had normal histological features. The free BVC, NLC-L-BVC, and dacarbazine groups showed foci of moderate to severe inflammatory infiltrates. Thus, histology results do not indicate any substantial changes in any group studied.

### 3.5 Animal Survival

Animal survival was determined as the time (in days) between tumor induction and the animal’s death. In accordance with a previously established protocol,^5^ animal euthanasia was performed in a controlled and supervised manner to relieve pain whenever clinical signs of any indication of pain or suffering (such as lethargy or severe weight loss) were observed. Figure 9A compares the cumulative survival of the animals in the experimental groups as a function of the length of the experiment (22 days in total).

**Figure 9:**
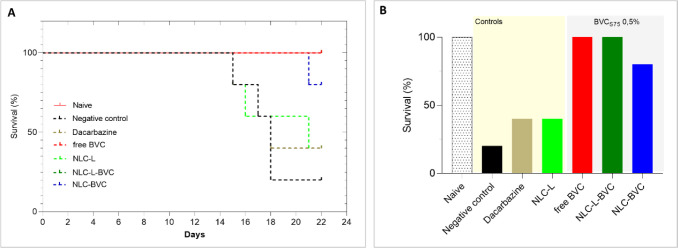
(A) Animal survival after melanoma induction. The survival rate was 100% in the naive, free BVC, and NLC-L-BVC groups; 80% in the NLC-BVC group (1 animal died); 40% in the dacarbazine and NLC-L groups (3 animals/group died); and 20% in the negative control group (4 animals/group died). (B) Percentage of survival across treatments, indicating higher survival odds for animals treated with BVC_S75_ (free or encapsulated).

The negative control group (untreated tumor) exhibited the lowest survival rate (20%). After treatment with dacarbazine and NLC-L, the survival rate was 40%. Possible contributing factors to the observed mortality in these groups include toxicity, as suggested by elevated CK-MB levels (Figure 7C) and metabolic alterations in the liver, as well as increased tumor growth during the post-treatment period (Figure 4A). Regarding dacarbazine, the treatment’s limited ability to extend overall survival may be attributed to chemoresistance.^117^

The highest survival rates (100%, 100%, and 80%) were observed in the groups treated with bupivacaine (free BVC, NLC-L-BVC, and NLC-BVC, respectively). Even though treatment with free BVC resulted in elevated serum CK-MB levels (Figure 7C) and no significant decrease in tumor growth (Figures 3 and 4), the animals in this group showed the highest survival rate, revealing the beneficial effect of the anesthetic on the cancer’s prognosis, in accordance with the literature.^3,6,91,118^ The high survival rate observed after encapsulation into the nanoparticles (NLC-L-BVC and NLC-BVC, Figure 9B) confirms that it is promoted by the anesthetic.

### 3.6 Final Considerations

This study evaluated the antitumor potential of two lipid nanoformulations prepared with natural and synthetic excipients and containing bupivacaine on melanoma tumor cells. The formulations consisted of BVC_S75_ (0.5%) encapsulated in NLC prepared with lavender essential oil (NLC-L-BVC) or Capryol 90 (NLC-BVC) as the liquid lipid. The *in situ* pharmacokinetic analysis confirmed that the nanoparticles prolonged, through sustained release, effective levels of BVC_S75_ in the tumor tissue, improving its pharmacological activity (inhibition of tumor volume) compared to the free drug. Tumor volume was lower in animals treated with NLC-L-BVC or NLC-BVC than in those treated with the reference drug dacarbazine, and the formulations were effective in inhibiting tumor growth during and after treatment. The systemic parameters analyzed (animal weight, biochemical, and NMR metabolomics) revealed no clinically relevant toxicity for the formulations and confirmed that encapsulated BVC_S75_ improved the prognosis while showing no signs of systemic toxicity. Additionally, higher survival rates in the immediate post-treatment period were observed in all treatments with BVC.

## 4 Conclusions

The obtained data evidenced a better prognosis when BVC_S75_ was used in the treatment of melanoma, promoting higher survival rates compared to the control groups (without BVC_S75_). Encapsulation of BVC_S75_ in NLC promoted its sustained release at the site of action and, thus, greater antitumor effectiveness in the local treatment of melanoma. Therefore, BVC-in-NLC can offer a possible advancement in oncologic therapy with the benefit of greater local action (anesthetic and antitumor effect) and improved prognostic outcomes without systemic toxicity. Unfortunately, the association of LO and BVC_S75_ in lipid nanoparticles did not result in a clear synergy for the local treatment of melanoma. Although NLC-L (without BVC_S75_) displayed activity during the chemotherapy treatment of the primary tumor, its post-treatment outcomes (tumor volume, histopathological analysis, and systemic effects) were suboptimal. In conclusion, natural (NLC-L-BVC) and synthetic (NLC-BVC) formulations were found to be equally effective in the treatment of melanoma.

## Supplementary Material



## Notes

Tumor microdialysis in an animal model (Figure S1); additional information regarding tumor evolution during treatment (Figure S2); histological analyses of the primary tumor in animals of the naive group (Figure S3A), negative control group (Figure S3B), and groups that received: dacarbazine (Figure S3C), free BVC (0.5%) (Figure S3D), NLC-L-BVC (Figure S3E), NLC-BVC (Figure S3F) or NLC-L (Figure S3G); tissue-to-body weight coefficients (for the liver, heart, lungs, kidneys, and spleen) after treatment (Figure S4); representative ^1^H NMR spectrum of the aqueous liver extract (Figure S5); histological images of organs (spleen, liver, lungs, and kidneys) from animals in the naive group and those treated with 0.9% NaCl, dacarbazine, free BVC, NLC-L, NLC-L-BVC, or NLC-BVC, stained with H&E (Figure S6), certificates for the *in vivo* experimental protocols (Figure S7) (PDF)
