# Supplementary material for: Enantiomeric Excess Bupivacaine in a Lavender Oil NLC Tested in a Melanoma Model: Prolonged Release and Anticancer Effect
Source: Mol Pharm. 2025 May 1;22(6):3351–65. doi: 10.1021/acs.molpharmaceut.5c00254 (PMC12135038; doi:10.1021/acs.molpharmaceut.5c00254)
Supplement: Supplementary file 1 [file mp5c00254_si_001.pdf]

SUPPORTING INFORMATION

ENANTIOMERIC EXCESS BUPIVACAINE IN A LAVENDER OIL NLC TESTED IN A MELANOMA MODEL: PROLONGED RELEASE AND ANTICANCER EFFECT

Gabriela Geronimo[1], Gustavo H. Rodrigues da Silva[2*], Ludmilla D. de Moura[1], Fabíola V. de Carvalho[1], Talita C. Mendonça[1], Laura B. Olivo[3], Bibiana Verlindo de Araújo[3], Teresa Dalla Costa[3], Luccas Lavareze[4], Fernanda V. Mariano[4] and Eneida de Paula [1*]

1 Department of Biochemistry and Tissue Biology, Institute of Biology, University of Campinas—UNICAMP, Campinas 13083-862, SP, Brazil.

2 Brazilian Biosciences National Laboratory, Brazilian Center for Research in Energy and Materials, Campinas 13083-970, SP, Brazil.

3 Pharmaceutical Sciences Graduate Program, Faculty of Pharmacy, Federal University of Rio Grande do Sul—UFRGS, Porto Alegre 90610-000, RS, Brazil.

4 Department of Pathology, Faculty of Medical Sciences, UNICAMP, Campinas 13083-888, SP, Brazil.

* Correspondence: depaula@unicamp.br and gustavohrs@gmail.com

1. Intratumor microdialysis in the tumor interstitial space of C57BL/6J mice

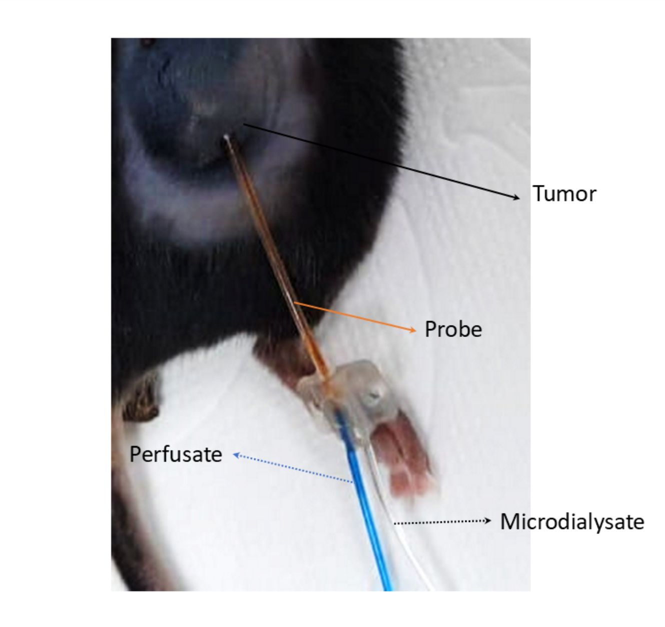

Figure S1. Tumor microdialysis in an animal model. Intratumor microdialysis and quantification of bupivacaine S75:R25 (BVC_{S75}) in the tumor interstitial space of C57BL/6J mice.

2. Effect of treatments in the primary tumor

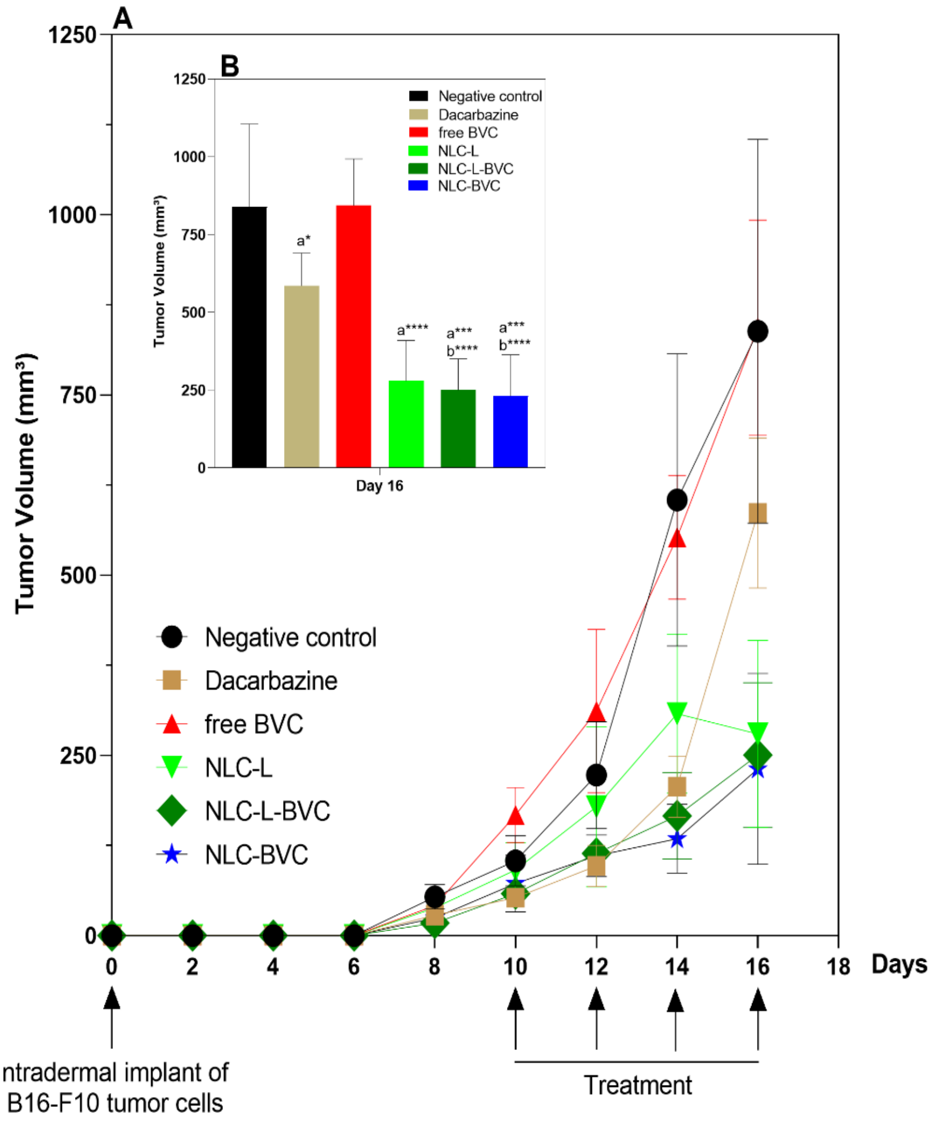

Figure S2. Tumor growth during treatment. (A) Evolution of tumor volume for animals treated with negative control (0.9% NaCl), positive control (dacarbazine), free BVC (0.5%), lavender oil formulation (NLC-L), or formulations containing 0.5% BVCS75 (NLC-L-BVC and NLC-BVC). (B) Tumor volume for each experimental group at day 16 (last day of treatment). Data represent mean ± SD (n = 5). Statistical analysis: two-way ANOVA plus post-hoc Tukey. a = in comparison to the negative control; b = compared to free BVC. * $p < 0.05$; *** $p < 0.001$; **** $p < 0.0001$.

3. Histological analysis of the primary tumor

The animals in the Naive group (Fig. S3A) had normal skin, without tumor. The histology of these animals showed the presence of well-defined layers (epidermis, dermis, and hypodermis), with hair follicles, hairs with melanin, sebaceous glands and adipose tissue with the usual characteristics. The melanocytes, located in the basal layer, had clear cytoplasm and a centralized or peripheral nucleus, with low melanin production.

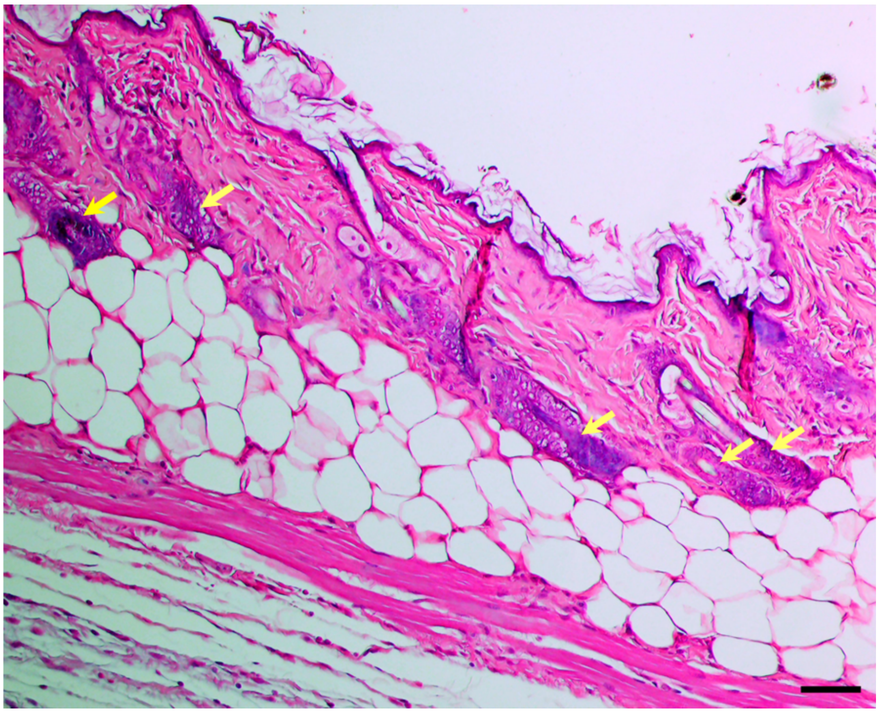

Figure S3A. Histopathological section of excised health skin (H & E staining). Scale bar = 100 µm, Magnification: 10×. Yellow arrows indicate hair follicles.

The tumor of the animals in the negative control group (untreated) (Fig. S3B) showed an aggressive tumor profile, recognized by extensive areas of necrosis and invasion of the underlying muscle and fat tissue, classified as Clark V (see methods).

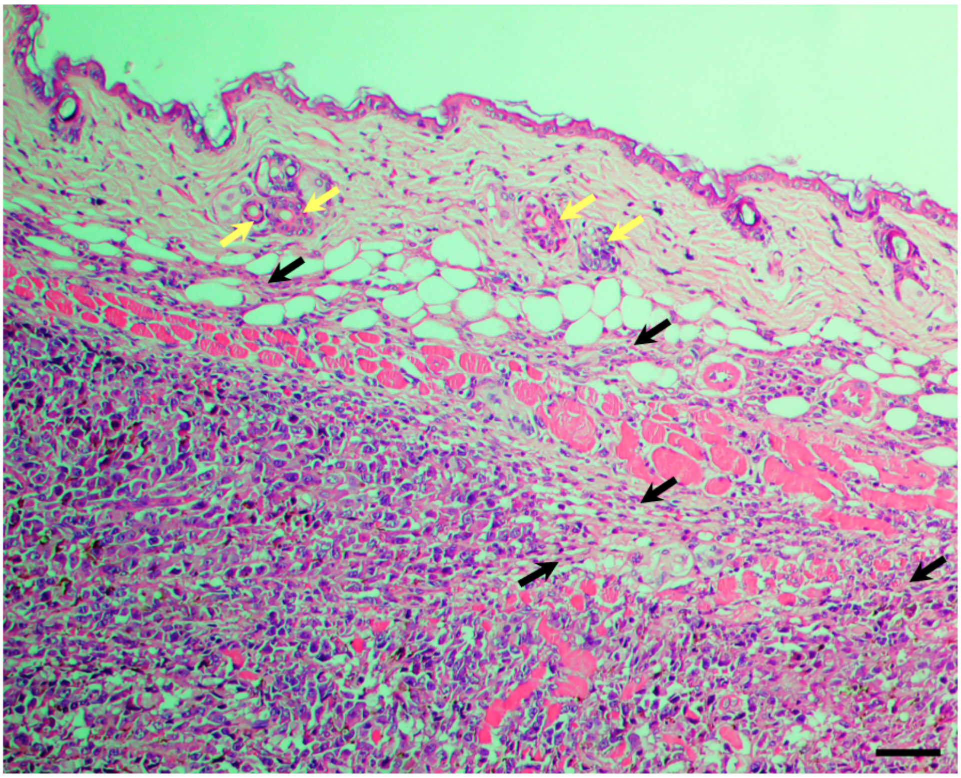

Figure S3B. Histopathological section of excised tumor without treatment – negative control (H & E staining). Scale bar = 100 µm, Magnification: 10×. Yellow arrows indicate hair follicles and the black arrows point to areas of necrosis.

In the group treated with dacarbazine (Fig. S3C), it was possible to observe areas of infarction, hemorrhage, edema, and the presence of lymph nodes unaffected by the melanoma, with focal clear cells (normal appearance), displaying Clark level IV.

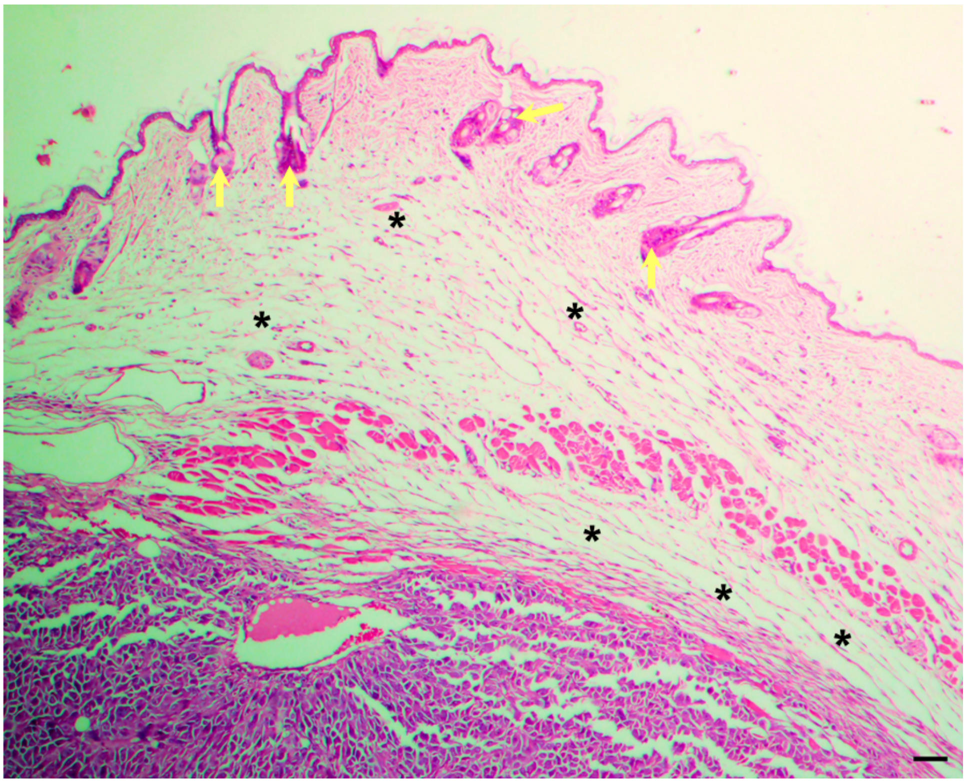

Figure S3C. Histopathological section of excised tumor after dacarbazine treatment (H & E staining). Scale bar = 100 µm, Magnification: 5×. Yellow arrows indicate hair follicles and the asterisks represent areas of edema.

The images from the free BVC group (Fig. S3D) showed proliferation of cancer cells arranged in a solid growth pattern. The adjacent tissue showed areas of vascular congestion, hemorrhage, inflammatory infiltrate, and fibrin. In this group, the animals' tumors showed larger areas of necrosis compared to the other groups, with a Clark IV level.

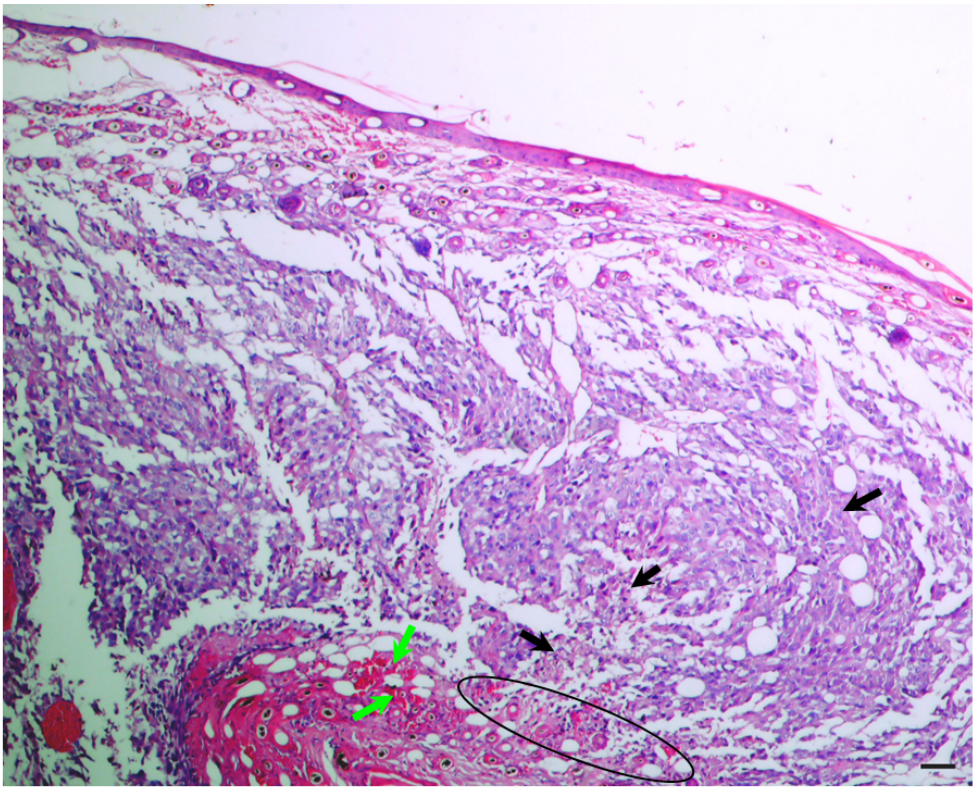

Figure S3D. Histopathological section of excised tumor after bupivacaine S75:R25 treatment (H & E staining). Scale bar = 100 µm, Magnification: 5×. The black arrows point to areas of necrosis, green arrows indicate hemorrhage, and the circled area shows inflammatory infiltrate.

In the slides of the animals treated with the nanoformulations containing lavender oil and BVC_{S75}, NLC-L-BVC (Fig. S3E), showed Clark IV level, with malignant cells arranged in a solid pattern, without disruption of the epithelium layer. There were also areas of intratumoral necrosis, with foci of melanocytic cells.

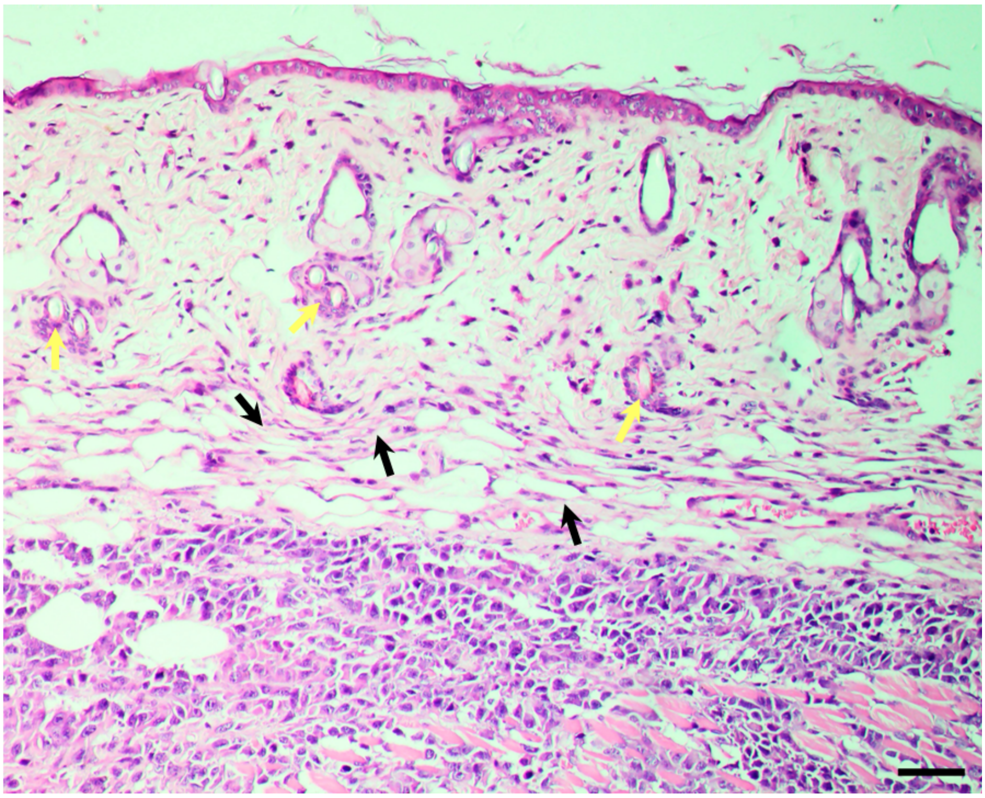

Figure S3E. Histopathological section of excised tumor after NLC-L-BVC treatment (H & E staining). Scale bar = 100 μm, Magnification: 10×. Yellow arrows indicate hair follicles and the black arrows point to areas of necrosis.

The NLC-BVC group (Fig. S3F) showed more areas of undifferentiation with spindle cells, solid tumor growth, intratumoral necrosis, edema, ulceration, moderate inflammatory infiltrate and invasion of the hypodermis, with a Clark V level.

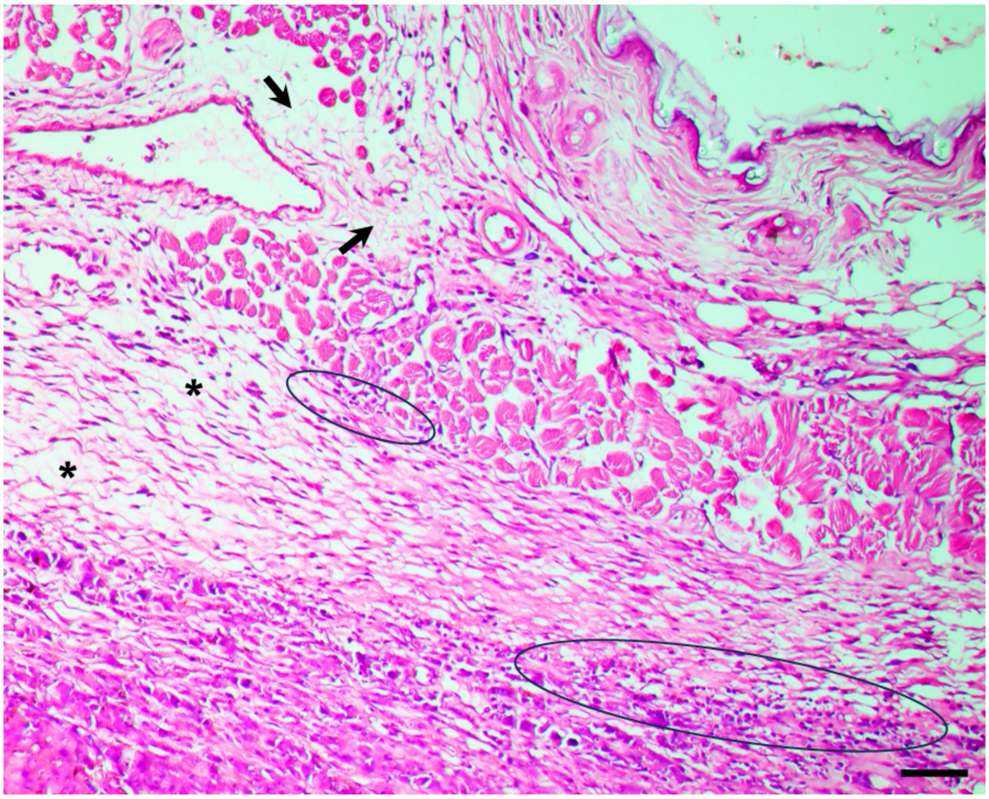

Figure S3F. Histopathological section of excised tumor after NLC-BVC treatment (H & E staining). Scale bar = 100 µm, Magnification: 10×. The black arrows point to areas of necrosis, the asterisks represent areas of edema, and the circled area indicate inflammatory infiltrate

In relation to the group treated with the control formulation (without BVC_{S75}, Fig. S3G): the tumor infiltrated the deep tissue in the NLC-L group, reaching the dermis and hypodermis (Clark classification level V).

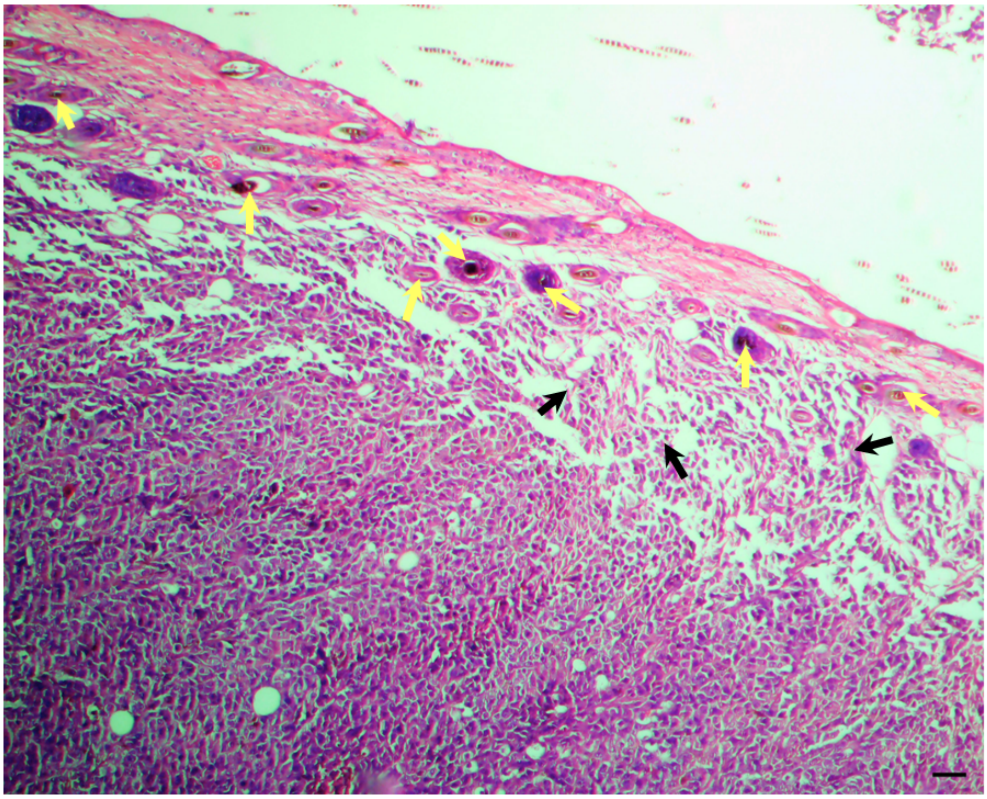

Figure S3G. Histopathological section of excised tumor after NLC-L treatment (H & E staining). Scale bar = 100 µm, Magnification: 5×. Yellow arrows indicate hair follicles and the black arrows point to areas of necrosis.

4. Systemic Evaluation and toxicological profile of the treatments

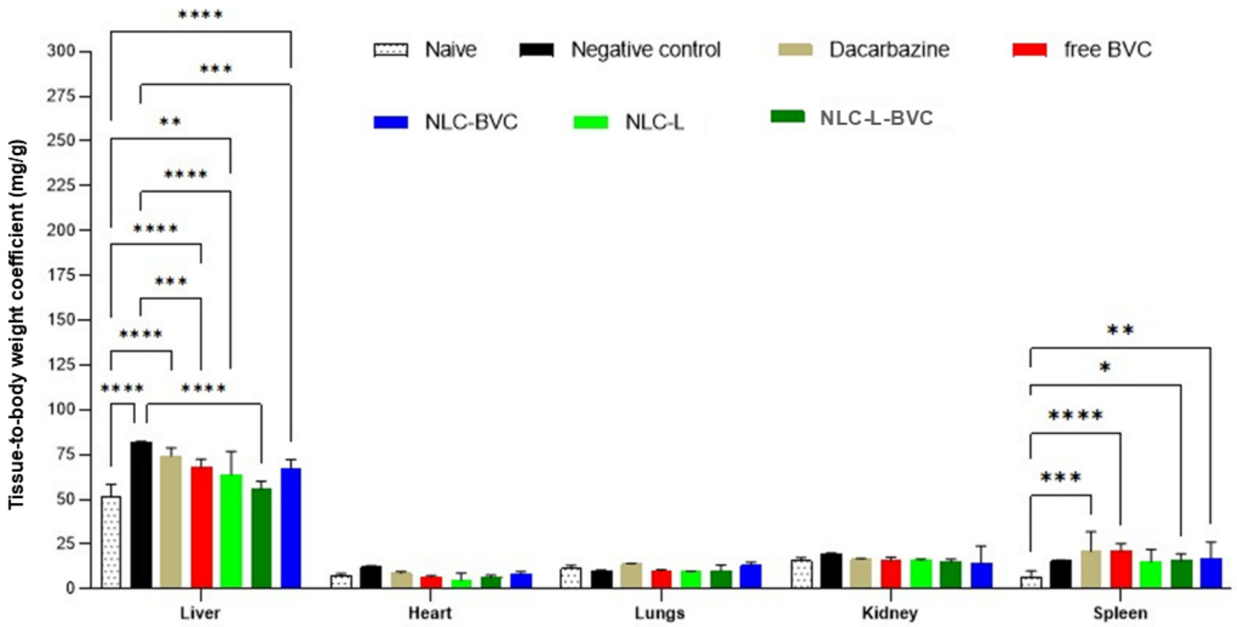

Figure S4. Tissue-to-body weight coefficients (for the liver, heart, lungs, kidneys, and spleen) after treatment. Statistical analysis: Two-way ANOVA plus post-hoc Tukey. * p < 0.05; ** p < 0.001; *** p < 0.001; **** p < 0.0001

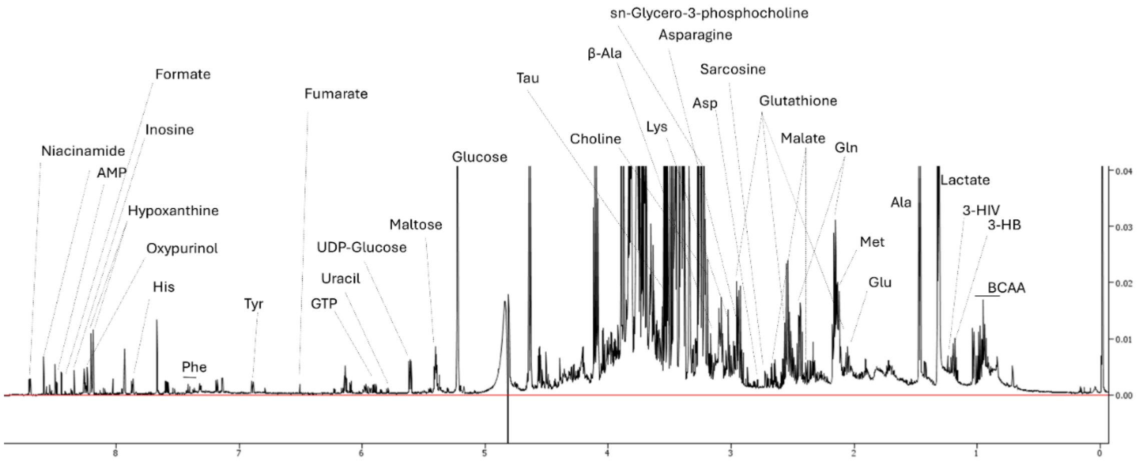

Figure S5. NMR spectrum of aqueous liver extract. Three-letter codes were used for amino acids; BCAA: branched chain amino acids (Leucine, Valine, Isoleucine); UDP-Glucose: Uridine diphosphate glucose, 3-HIV: 3- hydroxyvalerate; 3-HB: 3- hydroxybutyrate.

Figure S6 show the histological images of organs. The liver revealed hepatocytes with normal characteristics (polyhedral cells with well-distributed eosinophilic cytoplasm), delimited central lobular veins and no inflammatory infiltrate in all the treatment groups. The histological aspects of the kidneys were also within normal parameters: glomerular structures with the usual size, cellularity, capillary thickness, and width of Bowman's space, with no evidence of abnormalities or pathological alterations in any of the groups. The spleen showed histological characteristics considered normal in all the groups analyzed: in the white pulp, the presence of germinal centers formed by sheets of lymphocytes was observed, while in the red pulp, immune cells and blood capillaries were interspersed in extensive sheets.

Pulmonary evaluation revealed that the Naive, negative control, NLC-L and NLC-BVC groups had normal histological features, with well-defined lung parenchyma and airways, occasional presence of pulmonary macrophages and mild inflammatory infiltrate and fibrosis. The free BVC group showed focal areas of hemorrhage associated with a mild inflammatory infiltrate. The NLC-L-BVC and dacarbazine groups showed foci of moderate to severe inflammatory infiltrate.

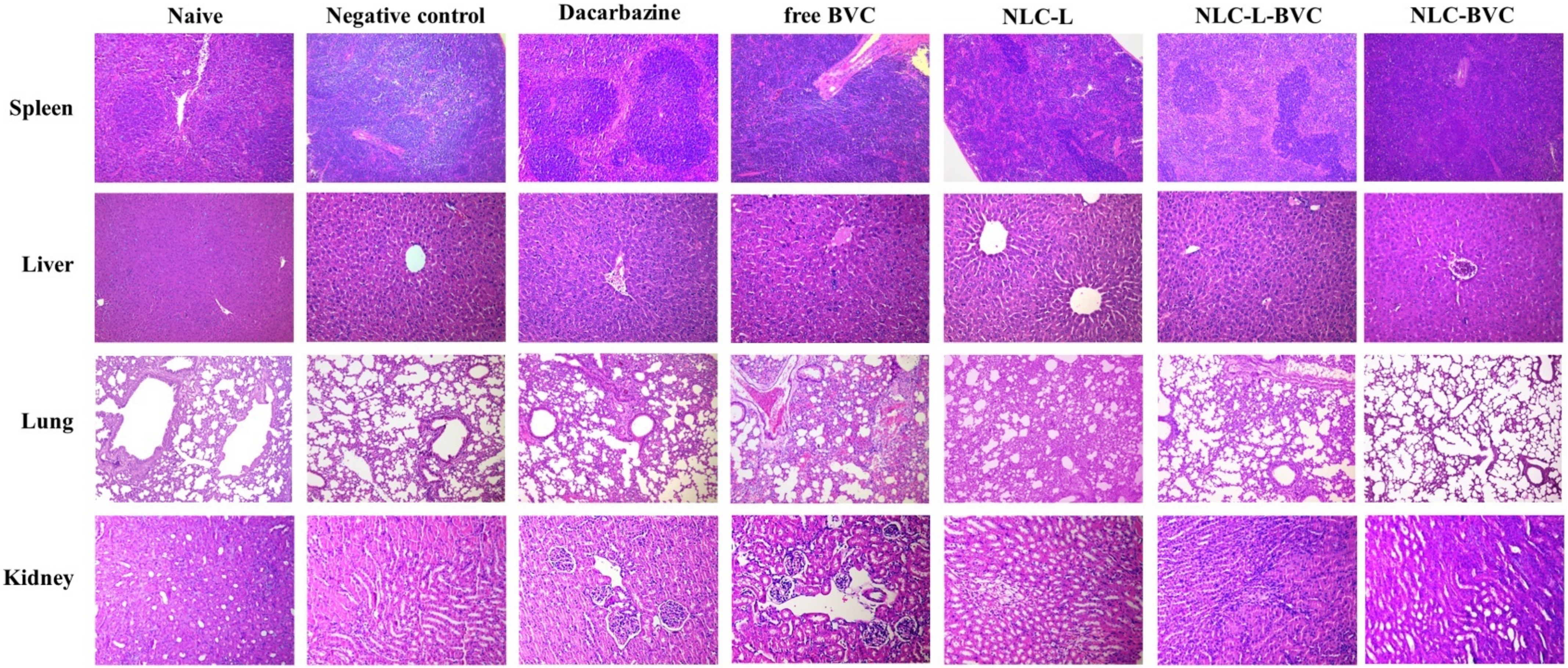

Figure S6. Histopathological sections of adult, female C57BL/6J (*Mus musculus*) mice in which (but the Naive group) a melanoma induced model was induced by B16-F10 (murine melanoma) cells. After euthanasia, the spleen, liver, lungs, and kidney of the animals treated with 0.9% NaCl, dacarbazine, free BVC, NLC-L, NLC-L-BVC or NLC-BVC were excised for the analysis (H & E staining), magnification: 5 × or 10 ×.

5. Approval certificates for the *in vivo* experimental protocols

UNICAMP

CEUA/UNICAMP

C E R T I F I C A D O

Certificamos que a proposta intitulada **Avaliação in vivo do efeito antitumoral em melanoma de bupivacaína encapsulada em carreadores lipídicos nanoestruturados preparados com óleo essencial de lavanda**, registrada com o nº **5736-1/2021**, sob a responsabilidade de **Prof. Dr. Eneida de Paula e Gabriela Geronimo**, que envolve a produção, manutenção ou utilização de animais pertencentes ao filo *Chordata*, subfilo *Vertebrata* (exceto o homem) para fins de pesquisa científica (ou ensino), encontra-se de acordo com os preceitos da **LEI Nº 11.794, DE 8 DE OUTUBRO DE 2008**, que estabelece procedimentos para o uso científico de animais, do **DECRETO Nº 6.899, DE 15 DE JULHO DE 2009**, e com as normas editadas pelo **Conselho Nacional de Controle da Experimentação Animal** (**CONCEA**), **tendo sido** aprovada pela **Comissão de Ética no Uso de Animais** da **Universidade Estadual de Campinas – CEUA/UNICAMP**, em reunião de **18/03/2021**.

**Finalidade:**	**( ) Ensino (X) Pesquisa Científica**
**Vigência do projeto:**	15/03/2021 a 15/03/2023
**Vigência da autorização para manipulação animal:**	18/03/2021 a 15/03/2023
**Espécie / linhagem/ raça:**	Camundongo isogênico / C57BL/6J
**No. de animais:**	10
**Idade/Peso:**	6.00 Semanas / 20.00 Gramas
**Sexo:**	10 Fêmeas
**Espécie / linhagem/ raça:**	Camundongo isogênico / C57BL/6J
**No. de animais:**	10
**Idade/Peso:**	6.00 Semanas / 20.00 Gramas
**Sexo:**	10 Fêmeas
**Espécie / linhagem/ raça:**	Camundongo isogênico / C57BL/6J
**No. de animais:**	10
**Idade/Peso:**	6.00 Semanas / 20.00 Gramas
**Sexo:**	10 Fêmeas
**Espécie / linhagem/ raça:**	Camundongo isogênico / C57BL/6J
**No. de animais:**	10
**Idade/Peso:**	6.00 Semanas / 20.00 Gramas
**Sexo:**	10 Fêmeas
**Espécie / linhagem/ raça:**	Camundongo isogênico / C57BL/6J
**No. de animais:**	10
**Idade/Peso:**	6.00 Semanas / 20.00 Gramas
**Sexo:**	10 Fêmeas
**Espécie / linhagem/ raça:**	Camundongo isogênico / C57BL/6J
**No. de animais:**	10
**Idade/Peso:**	6.00 Semanas / 20.00 Gramas
**Sexo:**	10 Fêmeas

CERTIFICADO CEUA nº 87/2021

**Espécie / linhagem/ raça:**	Camundongo isogênico / C57BL/6J
**No. de animais:**	10
**Idade/Peso:**	6.00 Semanas / 20.00 Gramas
**Sexo:**	10 Fêmeas
**Espécie / linhagem/ raça:**	Camundongo isogênico / C57BL/6J
**No. de animais:**	10
**Idade/Peso:**	6.00 Semanas / 20.00 Gramas
**Sexo:**	10 Fêmeas
**Espécie / linhagem/ raça:**	Camundongo isogênico / C57BL/6J
**No. de animais:**	10
**Idade/Peso:**	6.00 Semanas / 20.00 Gramas
**Sexo:**	10 Fêmeas
**Origem:**	CEMIB/Unicamp
**Biotério onde serão mantidos os animais:**	Biotério 1 - Área de Fisiologia e Biofísica, DBEF/IB /UNICAMP

A aprovação pela CEUA/UNICAMP não dispensa autorização a junto ao **IBAMA,SISBIO** ou **CIBio** e é **restrita** a protocolos desenvolvidos em biotérios e laboratórios da Universidade Estadual de Campinas.

Campinas, **28 de abril de 2021**.

Prof. Dr. Wagner José Fávaro
Presidente

Rosangela dos Santos
Secretária Executiva

IMPORTANTE: Pedimos atenção ao prazo para envio do relatório final de atividades referente a este protocolo: até 30 dias após o encerramento de sua vigência. O formulário encontra-se disponível na página da CEUA/UNICAMP, área do pesquisador responsável. A não apresentação de relatório no prazo estabelecido impedirá que novos protocolos sejam submetidos.

Documento assinado eletronicamente por **WAGNER JOSE FAVARO, COORDENADOR CEUA/UNICAMP**, em 30/04/2021, às 10:23 horas, conforme Art. 10 § 2º da MP 2.200/2001 e Art. 1º da Resolução GR 54/2017.

Documento assinado eletronicamente por **ROSANGELA DOS SANTOS, SECRETÁRIA EXECUTIVA CEUA/UNICAMP**, em 28/04/2021, às 15:52 horas, conforme Art. 10 § 2º da MP 2.200/2001 e Art. 1º da Resolução GR 54/2017.

A autenticidade do documento pode ser conferida no site: sigad.unicamp.br/verifica, informando o código verificador: **55CED6EB 84F04940 97A2CFF3 44DE3F0A**

UNICAMP SIGAD

Documento assinado. Verificar autenticidade em sigad.unicamp.br/verifica
Informar código 55CED6EB 84F04940 97A2CFF3 44DE3F0A

CERTIFICADO CEUA nº 46/2022

UNICAMP

CEUA/UNICAMP

CERTIFICADO

Certificamos que a proposta intitulada **Distribuição de bupivacaína encapsulada em CLN em tecidos alvos em modelo de melanoma murino utilizando microdiálise**, registrada com o nº **5940-1/2022**, sob a responsabilidade de **Prof. Dr. Eneida de Paula e Gabriela Geronimo, Gustavo H R da Silva**, que envolve a produção, manutenção ou utilização de animais pertencentes ao filo *Chordata*, subfilo *Vertebrata* (exceto o homem) para fins de pesquisa científica (ou ensino), encontra-se de acordo com os preceitos da **LEI Nº 11.794, DE 8 DE OUTUBRO DE 2008**, que estabelece procedimentos para o uso científico de animais, do **DECRETO Nº 6.899, DE 15 DE JULHO DE 2009**, e com as normas editadas pelo **Conselho Nacional de Controle da Experimentação Animal** (CONCEA), **tendo sido** aprovada pela **Comissão de Ética no Uso de Animais** da **Universidade Estadual de Campinas – CEUA/UNICAMP**, em reunião de **17/02/2022**.

**Finalidade:**	**( ) Ensino (X) Pesquisa Científica**
**Vigência do projeto:**	01/03/2022 a 01/12/2022
**Vigência da autorização para manipulação animal:**	17/02/2022 a 01/12/2022
**Espécie / linhagem/ raça:**	Camundongo isogênico / C57BL/6J
**No. de animais:**	10
**Idade/Peso:**	6.00 Semanas / 20.00 Gramas
**Sexo:**	10 Fêmeas
**Espécie / linhagem/ raça:**	Camundongo isogênico / C57BL/6J
**No. de animais:**	10
**Idade/Peso:**	6.00 Semanas / 20.00 Gramas
**Sexo:**	10 Fêmeas
**Espécie / linhagem/ raça:**	Camundongo isogênico / C57BL/6J
**No. de animais:**	10
**Idade/Peso:**	6.00 Semanas / 20.00 Gramas
**Sexo:**	10 Fêmeas
**Espécie / linhagem/ raça:**	Camundongo isogênico / C57BL/6J
**No. de animais:**	10
**Idade/Peso:**	6.00 Semanas / 20.00 Gramas
**Sexo:**	10 Fêmeas
**Espécie / linhagem/ raça:**	Camundongo isogênico / C57BL/6J
**No. de animais:**	10
**Idade/Peso:**	6.00 Semanas / 20.00 Gramas
**Sexo:**	10 Fêmeas
**Espécie / linhagem/ raça:**	Camundongo isogênico / C57BL/6J
**No. de animais:**	10
**Idade/Peso:**	6.00 Semanas / 20.00 Gramas
**Sexo:**	10 Fêmeas
**Espécie / linhagem/ raça:**	Camundongo isogênico / C57BL/6J
**No. de animais:**	10
**Idade/Peso:**	6.00 Semanas / 20.00 Gramas
**Sexo:**	10 Fêmeas
**Espécie / linhagem/ raça:**	Camundongo isogênico / C57BL/6J
**No. de animais:**	10
**Idade/Peso:**	6.00 Semanas / 20.00 Gramas
**Sexo:**	10 Fêmeas
**Origem:**	CEMIB
**Biotério onde serão mantidos os animais:**	Biotérios de Roedores da Bioquímica (bloco F), DBBT/IB/UNICAMP

A aprovação pela CEUA/UNICAMP não dispensa autorização a junto ao **IBAMA**,**SISBIO** ou **CIBio** e é **restrita** a protocolos desenvolvidos em biotérios e laboratórios da Universidade Estadual de Campinas.

Campinas, **15 de março de 2022**.

Prof. Dr. Wagner José Fávaro
Presidente

Rosangela dos Santos
Secretária Executiva

IMPORTANTE: Pedimos atenção ao prazo para envio do relatório final de atividades referente a este protocolo: até 30 dias após o encerramento de sua vigência. O formulário encontra-se disponível na página da CEUA/UNICAMP, área do pesquisador responsável. A não apresentação de relatório no prazo estabelecido impedirá que novos protocolos sejam submetidos.

Documento assinado. Verificar autenticidade em sigad.unicamp.br/verifica
Informar código 37A5EFC2 FA5645C5 A23E427E 7946F367

Documento assinado eletronicamente por **WAGNER JOSE FAVARO**, **PRESIDENTE DA CEUA/UNICAMP**, em 18/03/2022, às 15:15 horas, conforme Art. 10 § 2º da MP 2.200/2001 e Art. 1º da Resolução GR 54/2017.

Documento assinado eletronicamente por **ROSANGELA DOS SANTOS**, **SECRETÁRIA EXECUTIVA CEUA/UNICAMP**, em 17/03/2022, às 09:00 horas, conforme Art. 10 § 2º da MP 2.200/2001 e Art. 1º da Resolução GR 54/2017.

A autenticidade do documento pode ser conferida no site:
sigad.unicamp.br/verifica, informando o código verificador:
37A5EFC2 FA5645C5 A23E427E 7946F367

UNICAMP SIGAD

Figure S7. Ethical approval certificates for the use of animals in scientific research.
